# Quantitative Nanomechanical Analysis of Small Extracellular Vesicles for Tumor Malignancy Indication

**DOI:** 10.1002/advs.202100825

**Published:** 2021-08-02

**Authors:** Siyuan Ye, Wenzhe Li, Huayi Wang, Ling Zhu, Chen Wang, Yanlian Yang

**Affiliations:** ^1^ CAS Key Laboratory of Standardization and Measurement for Nanotechnology CAS Key Laboratory of Biological Effects of Nanomaterials and Nanosafety CAS Center for Excellence in Nanoscience National Center for Nanoscience and Technology Beijing 100190 P. R. China; ^2^ University of Chinese Academy of Sciences Beijing 100049 P. R. China; ^3^ Department of Chemistry Tsinghua University Beijing 100084 P. R. China; ^4^ State Key Laboratory of Natural and Biomimetic Drugs School of Pharmaceutical Sciences Peking University Beijing 100871 P. R. China; ^5^ Translational Medicine Center Chinese Institute for Brain Research (CIBR) Beijing 102206 P. R. China

**Keywords:** atomic force microscopy, cancer mechanobiology, extracellular vesicles, nanoindentation, nanomechanical property, tumor

## Abstract

The nanomechanical properties of tumor‐derived small extracellular vesicles (sEVs) are essential to cancer progression. Here, nanoindentation is utilized on atomic force microscopy (AFM) to quantitatively investigate the nanomechanical properties of human breast cancer cell‐derived sEVs at single vesicle level and explore their relationship with tumor malignancy and vesicle size. It is demonstrated that the stiffness of the sEVs results from the combined contribution of the bending modulus and osmotic pressure of the sEVs. The stiffness and osmotic pressure increase with increasing malignancy of the sEVs and decrease with increasing size of the sEVs. The bending modulus decreases with increasing malignancy of the sEVs and is lower in smaller sEVs. This study builds relationship between the nanomechanical signature of the sEV and tumor malignancy, adding information for better understanding cancer mechanobiology.

## Graphical abstract

The nanomechanical properties of the tumor‐derived small extracellular vesicles (sEVs) are quantitatively investigated by nanoindentation on atomic force microscopy (AFM). The stiffness of the sEVs comes from the combined contribution of the bending modulus and osmotic pressure of the sEVs. Increased stiffness and osmotic pressure and decreased bending modulus of the sEVs are associated with enhanced malignancy of the tumor.

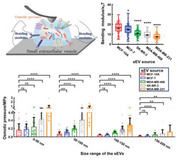

## 1 Introduction

Cancer mechanobiology plays significant roles in tumor progression and metastasis. The cells continuously adjust their behavior in response to the dynamic mechanical forces in the environment to maintain the physiological homeostasis of the tissue.^[^ ^1^, ^2^ ^]^ In response to the mechanical stimulation in the disrupted homeostasis during malignant transformation, the nanomechanical properties of the cells changed, which modulates the fundamental cell behavior and the microenvironment and ultimately leads to tumor invasion and metastasis.^[^ ^3^, ^4^ ^]^ The nanomechanical signatures of the tumor have become the diagnostic markers of tumorigenesis and tumor progression.^[^ ^5^ ^]^

Small extracellular vesicles (sEVs) are endosome‐derived membranous vesicles released by almost all the cells and are abundant in various body fluids. Since they carry the plasma membranes and the intracellular contents such as the cytoplasmic proteins and the nucleic acids from the source cells, their nanomechanical properties represent the ones of the source cells. Therefore, they can be taken as a desired model to study the nanomechanism of tumor.^[^ ^6^, ^7^ ^]^ The sEVs serve as the vehicles to transport the proteins, nucleic acids, and lipids to the recipient cells.^[^ ^6^ ^]^ The nanomechanical properties of the sEVs determine their behaviors in the fundamental processes such as intracellular communication,^[^ ^8^, ^9^ ^]^ diffusion, and transport in the extracellular matrix (ECM) and response to hemorheology during circulation,^[^ ^10^ ^]^ which ultimately decide tumor invasion and metastasis. To establish comprehensive communication between the nanomechanical signature of tumor‐derived sEVs helps to elucidate the role of the sEVs in tumor progression and metastasis, leading to the development of diagnostic markers of cancer. Moreover, the sEVs have attracted increasing attention as drug delivery vehicles due to their high biocompatibility, low immunogenicity, and their ability to cross the biological barriers.^[^ ^11^ ^]^ Nanomechanical characterization of the sEVs provides important information for optimizing the engineered sEVs to enhance drug delivery efficiency. However, nanomechanism characterization of the sEVs has been scarce. Several recent studies estimated the stiffness of tumor‐derived sEVs by measuring Young's modulus that represented the stiffness of the sEVs taking the sEVs as an integral elastic spherical model.^[^ ^12^, ^13^, ^14^ ^]^ Recently, several studies showed that the stiffness of the small vesicles resulted from the combined contribution from the nanomechanical properties (e.g., bending modulus and the osmotic pressure) which cannot be distinguished by Young's modulus alone.^[^ ^15^, ^16^, ^17^, ^18^ ^]^ The relationship between the individual nanomechanical property of tumor‐derived sEVs and malignant transformation is largely unknown. Atomic force microscopy (AFM) is well known for its high spatial resolution and has been used to characterize the dynamics of single EV and its interaction with biomolecules.^[^ ^19^, ^20^ ^]^ Nanoindentation on AFM records the nanomechanical response of single sEV at local points, from which the individual nanomechanical factors can be quantitatively extracted from the force indentation curves (FICs).^[^ ^15^, ^21^, ^22^ ^]^ The nanomechanical behavior of the sEVs during tip nanoindentation reflects the response of the sEVs to the nanomechanical stimulation at the physiological and pathological environment.

Here we quantitatively characterize the nanomechanical signatures of breast cancer‐derived sEVs at single vesicle level by nanoindentation on AFM and explore their correlation with tumor malignancy. We also compare the sEV subgroups with different sizes since the heterogeneous size of the sEV has been known to be associated with different molecular compositions and nanomechanical properties that determine the significance of sEV subpopulations as the diagnostic markers.^[^ ^13^, ^23^ ^]^ We demonstrate that the stiffness of the sEVs is determined by the combined contribution of the bending modulus and osmotic pressure of the sEVs. The bending modulus decreases with increasing malignancy of the sEVs and is lower in the sEVs with smaller sizes, while the stiffness and osmotic pressure increase with increasing malignancy of the sEVs and decrease with increasing sizes of the sEVs. These results shed light on the role of sEV mechanobiology in tumor progression and metastasis, showing the potential of sEV nanomechanical properties in evaluating tumor malignancy.

## 2 Results and Discussion

### 2.1 The Geometry of the Adsorbed sEVs Reflects Tumor Malignancy

**Figure 1:**
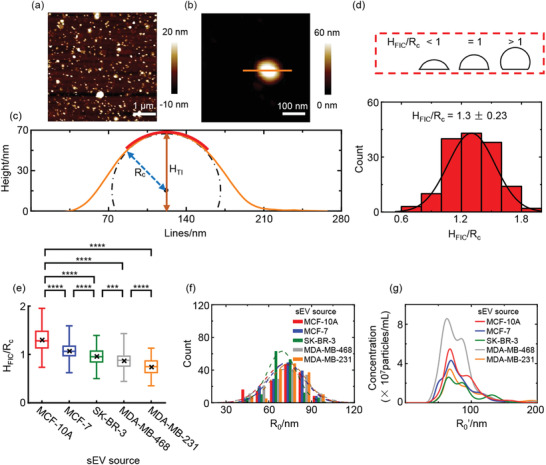
The size and geometry of the adsorbed sEVs. a) Large‐scale and b) high‐resolution AFM topographic images of sEVs derived from MCF‐10A. c) The contour of the sEV obtained by correcting the tip radius on the height profile along the orange solid line in (b). The red solid line and the black dashed line indicate the fitted circular arcs and the estimated shape of the sEVs, respectively, under the assumption that the sEVs form hemispherical caps on the substrate. d) Determination of the shape and geometry of the adsorbed sEVs derived from MCF‐10A. The representative shapes of the adsorbed sEVs were illustrated in the red dotted box (defined by *H*_FIC_/*R*_c_). e) *H*_FIC_/*R*_c_ of the sEVs derived from the five cell lines. The means are presented as the crosses in the boxplots and quantitative data analyzed by one‐way ANOVA. Multiple comparison between the groups was performed using Bonferroni method. Statistical significance was set at a level of ***p < 0.001 and ****p < 0.0001. f) The estimated radius (*R*_0_) and g) the initial radius (*R*_0_′) of the sEVs from different cell lines: *R*_0_ = 71 ± 14, 72 ± 12, 69 ± 10, 72 ± 15, 74 ± 12 nm and *R*_0_′ = 77 ± 39, 72 ± 35, 75 ± 34, 72 ± 37, 75 ± 35 nm, respectively. The dashed lines show the Gaussian fits. *R*_0_′ is determined by NTA. All values are means ± s.d. (*N* = 150 sEVs in each cell line).

We investigated the sEVs derived from the normal human mammary epithelial cell line MCF‐10A and four human breast cancer cell lines with increasing malignancy: MCF‐7 (luminal A), SK‐BR‐3 (human epidermal growth factor receptor 2‐overexpression), MDA‐MB‐468 (basal epithelial), and MDA‐MB‐231 (basal mesenchymal).^[^ ^24^, ^25^ ^]^ The sEVs purified from the sEV‐free cell culture were adsorbed on poly‐l‐lysine‐coated mica before AFM imaging.^[^ ^26^ ^]^ Topographic images revealed that the sEVs were in a spherical cap‐like morphology (**Figure** **1**a,b), characteristic of the morphology of the vesicles adsorbed on the surface.^[^ ^27^ ^]^ After correcting the radius of the tip on the height profiles of the sEVs, the contours of the sEVs were fitted with the curvature arcs (red solid lines in Figure 1c) from which the estimated profiles (dashed lines in Figure 1c) of the adsorbed sEVs were depicted under the assumption that the sEVs formed hemispherical caps on the substrate. The radius of the estimated profiles (*R*_c_) were taken as the radius of the adsorbed sEV (Figure 1c). As the sEVs were soft materials, *R*_c_ might reflect the deformation of the sEVs caused by tip indentation (Figure S1, Supporting Information). We determined the height of the sEVs above the substrate without deformation by analyzing the FICs.^[^ ^28^ ^]^ We selected the point with the indentation of “0” on the FIC as the “zero‐force contact point.” The height from the zero‐force contact point to the maximum indentation in the FIC (*H*_FIC_) could be identified as the real height of the sEV above the substrate without deformation (Figure S2a, Supporting Information). We found that *H*_FIC_ was 19 ± 6 nm (s.d., *N* = 150 sEVs), higher than the height of the sEVs measured from the topography images (*H*_TI_, Figure S2b, Supporting Information). We can therefore calculate the imaging force‐induced deformation (Δ*ε*_apex_, defined as the difference between *H*_FIC_ and *H*_TI_) of the sEVs from which *R*_c_ can be determined (Figure S2c, Supporting Information). The geometry of the adsorbed sEV was determined by the value of *H*_FIC_/*R*_c_: =1, hemispherical; <1, sub hemispherical; >1, super hemispherical (Figure 1d). The average *H*_FIC_/*R*_c_ of tumor‐derived sEVs was much smaller than the one from the normal cell line MCF‐10A, and declined with increasing malignancy of the source cells, indicating that the highly malignant sEVs suffered more deformation on the substrate, which reflected possible different nanomechanical properties of the high‐malignant sEVs (Figure 1e). We calculated the initial radius (*R*_0_) of the sEVs from the surface area of the sEVs that was approximated from the AFM topographic images assuming that the surface area of each sEVs was constant after adherence on the substrate.^[^ ^29^ ^]^ We found that the estimated *R*_0_ of the sEVs from different cell lines were very similar (69 ~≈ 74 nm, Figure 1f), in accordance with the results from nanoparticle tracking analysis (NTA) that also showed similar radii (*R*_0_′, 72 ≈ 76 nm, Figure 1g) of the sEVs from different cell lines though the values were slightly lower than the calculated ones, indicating that the different geometry of the adsorbed sEVs came from the deformation of sEVs on the substrate, rather than the different sizes of the sEVs.

### 2.2 Mechanical Behavior of the sEV

**Figure 2:**
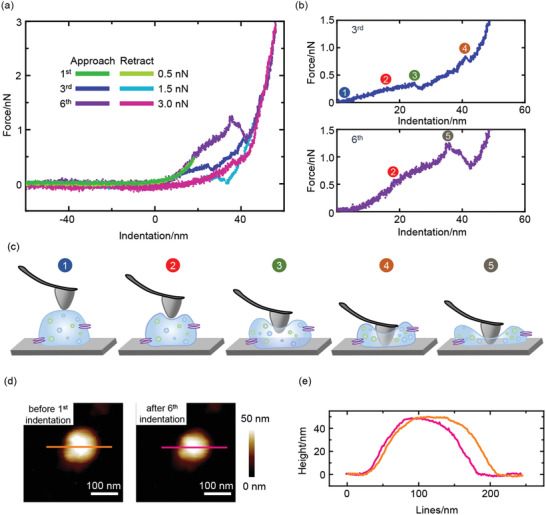
The nanomechanical behavior of the sEVs. a) Representative FICs showing the sequential indentations on the same sEV with increasing forces. The dark colors represent the approach curve and the light colors represent the retract curve. b) The enlarged approach curves of the third (1.5 nN) and sixth (3.0 nN) indentations. The numbers in the circles indicate the nanomechanical behavior of sEV at different stages. c) Scheme illustration of the nanomechanical response of the sEV against tip indentation from steps 1–5 in (b): 1, nanoindentation starts; 2, inward tether force forms; 3, tip penetrates the upper membrane of the sEV; 4, tip penetrates the lower membrane of the sEV; 5, the upper and lower membranes of the sEV are pushed together. d) AFM topographic images and e) the corresponding height profiles of the same sEV before the first indentation (orange) and after the sixth indentation (pink) through the maximum height. The sEV was derived from MCF‐10A cells.

We utilized nanoindentation to investigate the individual nanomechanical property of the sEVs. Sequential nanoindentations were performed at the center point of the sEVs with the force increased from 0.5 to 3 nN at 0.5 nN intervals at a loading rate of 250 nm s^−1^. When the applying force was low (0.5 and 1.0 nN in the first and second indentations, **Figure** **2**a; and Figure S3, Supporting Information), the complete overlap was observed between the approach and retract curves, indicating that the initial mechanical response of the sEV was fully elastic. As the force increased (1.5, 2.0, and 2.5 nN in the third, fourth, and fifth indentations, Figure 2a–c; Figure S3, Supporting Information), after a linear stage indicating the deformation of the sEV, a plateau appeared in the approach curve, indicating decreased force response at this stage. This might be due to the formation of inward tether force between the membrane and the tip.^[^ ^15^ ^]^ After the plateau, a linear relationship appeared again between the force and indentation as the resistant force caused by further indentation and sEV deformation overcame the inward tether force. Two breaks were observed after the plateau in the approach curve, suggesting that the tip penetrated the upper and lower membranes of the adsorbed sEV. When the force further increased (3.0 nN in the sixth indentation, Figure 2a,b), only one break was observed in the approach curve, which might be because that the upper and lower membranes were pushed together and penetrated by the tip. However, after sequential indentations, the height and shape of the sEVs remained almost unchanged (Figure 2d,e), suggesting that after transient sEV deformation and membrane penetration, the lipid membrane of the sEV self‐repaired as a result of the fluidity and dynamic organization of the lipids in the membrane, and the integrity and elasticity of the sEV hence remained. This was consistent with the previous observation with the liposomes that nanoindentations at the same position would not cause permanent damage nor affect the overall height of the liposome.^[^ ^15^ ^]^ In very few cases, after a large irreversible break that indicated membrane penetration and strong sEV deformation, the slope of the linear force response stage in the FICs significantly declined (Figure S4a, Supporting Information), suggesting that the sEVs might be ruptured and the enclosed solutes were released, resulting in decreased stiffness of the sEVs and the weakened force response. This was supported by the topographic images revealing the changed geometry and decreased height of the sEVs after sequential indentations (Figure S4b,c, Supporting Information). However, most of the time, no obvious deformation of the sEVs was found after sequential indentations (Figure S4d, Supporting Information), suggesting no permanent damage of the sEVs by nanoindentation.

### 2.3 The Combined Contribution of Bending Energy and Osmotic Pressure to the Stiffness of the sEVs

**Figure 3:**
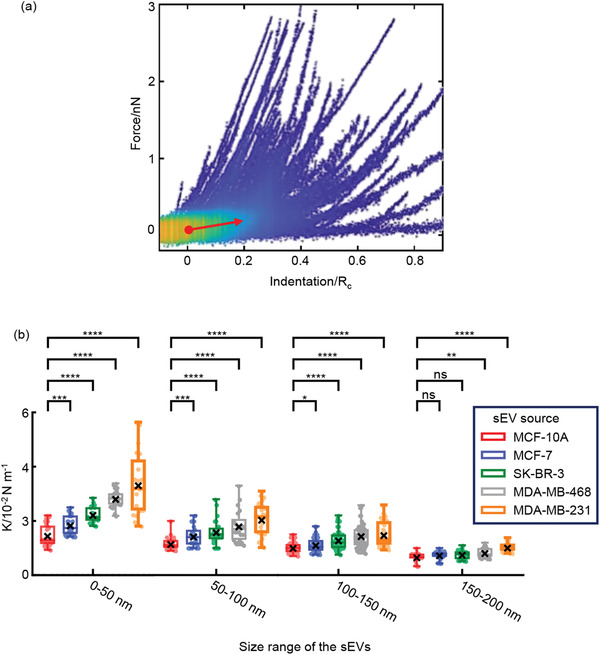
The stiffness of the sEVs. a) The density plot shows the FICs taken from 45 MCF‐10A‐derived sEVs. Six FICs per sEV were selected for calculation. Colors indicate the density of the data points at the typical spot where yellow represents high density while blue represents low density. FICs are displayed until their first discontinuities. The effective linear response region was determined from the average FIC using home‐built MATLAB software. The stiffness of the sEV is determined from the slope of the fitting (red arrow) of the obtained linear response region (0.05–0.2*R*_c_). b) The stiffness of the sEVs with different size ranges and from different source cell lines. Box plots in which the median is marked by the line in the box, box limits indicate upper and lower quartiles and whiskers indicate 1.5× interquartile range. Black crosses indicate mean as determined from the data sets. The scattered points indicate the specific value of each sEV. The number of sEVs included for the analysis are (in the order of MCF‐10A, MCF‐7, SK‐BR‐3, MDA‐MB‐468, and MDA‐MB‐231): 0–50 nm: *N* = 30, 26, 24, 26, 23; 50–100 nm: *N* = 39, 28, 24, 22, 31; 100–150 nm: *N* = 57, 46, 49, 52, 57; 150–200 nm: *N* = 23, 33, 28, 25, 24. (*p < 0.05, **p < 0.01, ***p < 0.001, ****p < 0.0001, ns not significantly, Two‐tailed *t*‐test).

We calculate the stiffness of the sEVs by fitting the linear force response region (0.05–0.2 *R*_c_, **Figure** **3**a). We found that the stiffness of the sEVs decreased with increasing size of the sEVs (Figure 3b; Table S1, Supporting Information). This was as expected as the sEVs with a smaller curvature radius had higher bending energy that contributed to the rigidity of the sEVs.^[^ ^30^ ^]^ The stiffness of the sEVs increased with the malignancy of the source cells (Figure 3b; Table S1, Supporting Information). Since studies have demonstrated that the nanoparticles with more rigidity are easier to enter the cells via membrane wrapping compared to the soft ones,^[^ ^31^ ^]^ we assume that the highly malignant sEVs might be easier to enter the recipient cell. This supported the more severe tumor invasion and metastasis in the highly malignant tumors.

We next intended to investigate the inherent characteristics that contributed to the stiffness of the sEVs. Previous studies reported that the initial linear response in the force‐indentation curves of the lipid membrane that was associated with a classic spring‐like behavior was decided by the inherent characteristics such as bending modulus and stretch modulus.^[^ ^32^, ^33^ ^]^ Since the applied force by tip indentation is perpendicular to the sEV, tensile modulus could be ignored, and bending energy might be the main nanomechanical property contributing to sEV stiffness.^[^ ^21^, ^34^ ^]^ In this case, the stiffness of the sEV could be defined as the bending energy per square of sEV radius, that is *K* = *κ*/*R*_c_^2^. As phosphatidylcholine (PC) is the predominant lipid component of the membrane in most sEVs (~≈46–89%),^[^ ^13^ ^]^ we used the bending modulus of a PC‐composed lipid bilayer (10–30 *κ*_b_ *T*) as the approximate bending modulus of the sEVs.^[^ ^35^ ^]^ The stiffness of the sEVs with the size ranging from 30 to 200 nm was estimated to be on the order of 10^–3^ to 10^–6^ N m^−1^. However, the actual stiffness calculated from the FICs was in the magnitude of 10^–2^ N m^−1^ (Figure 3b), one to four orders higher than the theoretical value, indicating that bending energy alone was far from being the only contribution to the stiffness of the sEVs.

It has been known that an outward tether is formed during the retraction of the tip as a result of the adhesion between the tip and the vesicle membrane, and the outward tether force (*F*_t_) is determined by the bending modulus (*κ*) and membrane tension (*σ*) of the vesicle following Equation (1).^[^ ^36^ ^]^

(1) $$F_{t}=2\pi \sqrt{2\sigma \kappa }$$

As the membrane tension mainly comes from the osmotic pressure across the membrane, the osmotic pressure (ΔΠ) can be determined by “Young–Laplace” equation.^[^ ^37^ ^]^

(2) $$\Delta \Pi =2\sigma /R_{c}$$

The adhesion‐induced osmotic pressure (ΔΠ) can be therefore calculated from the tether force (*F*_t_) by equation.

(3) $$\Delta \Pi ={\left(F_{t}/2\pi \right)}^{2}/\kappa R_{c}$$

**Figure 4:**
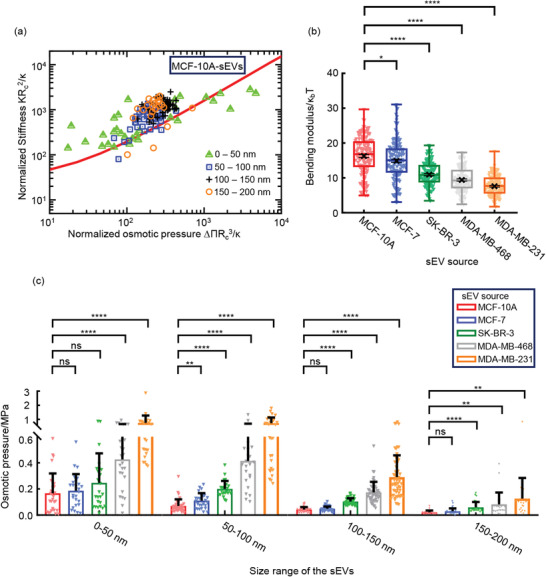
The bending modulus and osmotic pressure of the sEVs. a) Estimation of the bending modulus of the sEVs. The osmotic pressure and the stiffness are both normalized to make them dimensionless. Markers represent the bending modulus from MCF‐10A‐derived sEVs with different size ranges (0–50 nm, *N* = 30; 50–100 nm, *N* = 39; 100–150 nm, *N* = 57; 150–200 nm, *N* = 23). The theoretically predicted curve is shown in red. b) The bending modulus of the sEVs derived from different cell lines. Box plots in which the median is marked by the line in the box, box limits indicate upper and lower quartiles and whiskers indicate 1.5× interquartile range. The black crosses and the error bars indicate the mean and s.e.m. determined by bootstrapping (1000 repetitions). c) The osmotic pressure of the sEVs with different size ranges and from different source cells. Histogram bars indicate means, and error bars indicate standard deviation (s.d.). The scattered points indicate the specific value of each sEV. The number of sEVs included for the analysis are (in the order of MCF‐10A, MCF‐7, SK‐BR‐3, MDA‐MB‐468, and MDA‐MB‐231): 0–50 nm: *N* = 30, 26, 24, 26, 23; 50–100 nm: *N* = 39, 28, 24, 22, 31; 100–150 nm: *N* = 57, 46, 49, 52, 57; 150–200 nm: *N* = 23, 33, 28, 25, 24. (*p < 0.05, **p < 0.01, ***p < 0.001, ****p < 0.0001, ns not significantly, Two‐tailed *t*‐test).

According to a recent model describing the nanomechanical behavior of the vesicles based on Canham–Helfrich theory, the bending modulus of the liposomal small vesicles can be evaluated from the stiffness (*K*), radius (*R*_c_), and osmotic pressure (ΔΠ) of the vesicles.^[^ ^15^, ^38^ ^]^ By calculating the inflection point of the sEV where the inward tether force formed, we demonstrated that Canham–Helfrich model was suitable to describe the nanomechanical behavior of the sEVs considering the fluidity of the membrane (Figure S5, Supporting Information). After normalization to make the osmotic pressure and the stiffness dimensionless, a relationship can be generated from the normalized osmotic pressure (ΔΠ*R*_c_^3^/*κ*) and the normalized stiffness (*KR*_c_^2^/*κ*) (**Figure** **4**a; Figure S6, Supporting Information). As the adhesion‐induced osmotic pressure is dominant over the one from the additional indentation,^[^ ^39^ ^]^ the osmotic pressure here can be taken as the adhesion‐induced osmotic pressure determined from the outward tether force that can be calculated form the two linearly fitted regions in the retrace curve of the FIC (Figure S7, Supporting Information). The bending modulus was the only unknown parameter in this generated relationship. The sum of the squared Euclidian distance between the logarithm of the theoretically predicted curve and the experimental values:

(4) $$\sum_{i=1}^{n}\min_{j}\log{\left(\Delta \Pi_{i}R_{c_{i}}^{3}\kappa^{-1}/X_{j}\right)}^{2}\log{\left(K_{i}R_{c_{i}}^{2}\kappa^{-1}/Y_{j}\right)}^{2}$$

was minimized to obtain the bending modulus *κ* of the sEVs.^[^ ^38^ ^]^ We found that when the osmotic pressure was low, the contribution of the osmotic pressure on the stiffness of the sEVs was relatively low and the contribution of bending modulus was dominant. The bending modulus of the sEVs derived from MCF‐10A was determined to be ≈16 *κ*_b_ *T* (Figure 4a), similar to the value of the EVs derived from normal human RBCs that was ≈15 *κ*_b_ *T*.^[^ ^16^ ^]^ Tumor‐derived sEVs exhibited significantly lower bending modulus compared to the normal ones, and the bending modulus decreased with increasing malignancy of the source cells (Figure 4b). This might be due to the distinct composition of the membrane lipids in the malignant sEVs that reduced the bending rigidity of the sEVs via the altered lipid charge, hydrocarbon chain length, and unsaturation degree.^[^ ^35^, ^40^, ^41^ ^]^ On the other hand, proteomic analyses have revealed different protein profiles in tumor‐derived sEVs and have demonstrated significantly more proteins expressed in the sEVs from the highly malignant breast cancer compared to the low malignant one and the normal controls at both cellular and patient levels.^[^ ^42^, ^43^ ^]^ As the membrane proteins have been known to soften the membranes by inducing membrane thinning,^[^ ^44^, ^45^ ^]^ affecting the local organization and curvature of the lipid membrane or decoupling the lipid bilayer leaflets,^[^ ^46^, ^47^, ^48^ ^]^ it is reasonable to assume that the abundant membrane proteins in the malignant sEVs impacted on the membrane structure and reduced the bending modulus of the sEVs. We observed higher bending modulus of the sEVs with increasing size ranges, though no obvious variation was found in the sEV subgroups with the size larger than 100 nm (Figure S8, Supporting Information). This difference might also come from the distinct lipid and protein composition in the small sEVs compared to the sEVs with larger sizes.^[^ ^13^ ^]^

We calculated the osmotic pressure from the tether force and the bending modulus by Equation (4) and found that the osmotic pressure decreased with increasing size of the sEVs (Figure 4c), consistent with the previous report showing that the increased osmotic pressure is the driving force for the shape transformation of the vesicles.^[^ ^49^ ^]^ For the sEVs with the same size range, the osmotic pressure increased with the malignancy of the source cells (Figure 4c), in the same trend as the stiffness of the sEVs with different malignancy (Figure 3b), suggesting that osmotic pressure provide the predominant contribution to the stiffness of the sEVs compared to the bending modulus.

### 2.4 Bending Modulus, Osmotic Pressure, and Stiffness of the sEVs for Evaluating Tumor Malignancy

**Figure 5:**
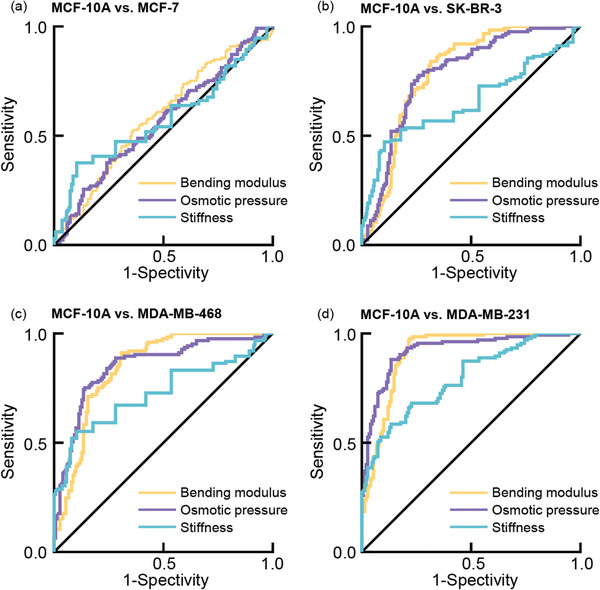
Receiver operating characteristic (ROC) analysis showing the discriminative efficacy of the bending modulus, osmotic pressure, and stiffness of the sEVs in distinguishing malignant and normal sEVs. ROC curve of the bending modulus (yellow line), the osmotic pressure (purple line), and the stiffness (blue line) in distinguishing sEVs derived from a) MCF‐10A (*N* = 149) and MCF‐7 cell line (*N* = 133), b) MCF‐10A (*N* = 149) and SK‐BR‐3 cell line (*N* = 125), c) MCF‐10A (*N* = 149) and MDA‐MB‐468 cell line (*N* = 125), and d) MCF‐10A (*N* = 149) and MDA‐MB‐231cell line (*N* = 135). The area under the ROC curve (AUC) of different group from (a) to (d): 0.5856, 0.5631, 0.5737; 0.7915, 0.7824, 0.6522; 0.8453, 0.8496, 0.7266; 0.9099, 0.9210, 0.7931 (bending modulus, osmotic pressure, and stiffness, respectively). The detailed data about s.d. error, 95% confidence interval, *p* value, and cut off value were displayed in the Table S2, Supporting Information.

We further performed receiver operating characteristic (ROC) analysis to evaluate the efficacy of the bending modulus, osmotic pressure, and stiffness of the sEVs in indicating tumor malignancy. We found that all three factors had high sensitivity and specificity in distinguishing mid to high‐malignant sEVs from the normal sEVs, though their discriminatory efficacy in discriminating low‐malignant sEVs from the normal ones was not satisfactory (MCF‐10A versus MCF‐7, area under the curve (AUC): 0.586, 0.563, 0.574; 95% confidence interval (CI): 0.519–0.652, 0.496–0.630, 0.505–0.642. MCF‐10A versus SK‐BR‐3, AUC: 0.792, 0.782, 0.652; 95% CI: 0.737–0.846, 0.727–0.837, 0.584–0.720. MCF‐10A versus MDA‐MB‐468, AUC: 0.845, 0.850, 0.727; 95% CI: 0.799–0.892, 0.802–0.897, 0.664–0.789. MCF‐10A versus MDA‐MB‐231, AUC: 0.910, 0.921, 0.793; 95% CI: 0.875–0.945, 0.889–0.953, 0.742–0.844. ROC analysis for bending modulus, osmotic pressure, and stiffness, respectively, **Figure** **5**a‐d; Table S2, Supporting Information). The three factors also had good performance in distinguishing high‐malignant and low‐malignant sEVs (Figure S9 and Table S2, Supporting Information). These results indicated the capability of the nanomechanical properties of the sEVs in evaluating tumor malignancy. Considering the size heterogeneity of the sEVs, we also performed ROC analysis in different sEV subgroups with different size ranges. We found that sEVs with different size ranges exhibited different performances in evaluating tumor malignancy (Figures S10–S12 and Tables S3–S5, Supporting Information), suggesting that the size of the sEVs should be taken into account when using the nanomechanical properties of the sEVs to evaluate tumor malignancy. We noticed that in some cases, stiffness of the sEV with smaller sizes (<100 nm) had better performance in discriminating sEVs with different malignancy, while osmotic pressure with smaller sizes (<50 nm) seemed to be less effective in discriminating sEVs with different malignancy (Figures S11,S12 and Tables S4,S5, Supporting Information). This might be because that the contact area was small between the tip (radius 20 nm) and the sEVs with smaller size (<50 nm), in which case the contribution from adhesion‐induced osmotic pressure was relatively small. Whereas for larger sEVs, tip indentation‐induced deformation from which we obtained the stiffness might be drowned to some extend by deformation of the sEVs on the substrate.

## 3 Conclusion

In summary, we have quantified the nanomechanical signatures of breast cancer‐derived sEVs and have demonstrated their correlation with tumor malignancy. The stiffness of the sEVs results from the combined contribution of the bending modulus and osmotic pressure. The stiffness and osmotic pressure increase with increasing malignancy of the sEVs and decrease with increasing sizes of the sEVs. The bending modulus decreases with increasing malignancy of the sEVs and is lower in smaller sEVs. These results showed the potential of sEV biophysical analysis in evaluating tumor malignancy, adding information to better understand the aggressiveness of cancer in terms of the mechanical properties of sEVs.

## 4 Experimental Section

### Cell Culture

All the breast cell lines were obtained from the American Type Culture Collection (Manassas, VA, USA). The following cell lines were used: MCF‐10A cells, MCF‐7 cells, MDA‐MB‐468 cells, SK‐BR‐3 cells, MDA‐MB‐231 cells. MCF‐10A cells were cultured in Mammary Epithelial Cell Basal Medium (Lonza; CC3151) supplemented with 0.4% bovine pituitary extract (Lonza; CC4009G), 0.1% human epidermal growth factor (Lonza; CC4017G), 0.1% hydrocortisone (Lonza; CC4031G), 0.1% GA‐1000 (Lonza; CC4081G), and 0.1% insulin (Lonza; CC4021G). The other four cells were cultured in DMEM (Gibco‐BRL) with 10% fetal bovine serum (GIBCO‐BRL) and 1% penicillin/streptomycin (GIBCO‐BRL). All the cells were incubated in a humidified atmosphere of 5% CO_2_/95% air at 37 °C. Subcultivation of the cell lines was performed using 0.25% trypsin and 5 × 10^−3^ m methylenediaminetetraacetic acid (EDTA) (Gibco‐BRL Co., MD, USA).

### sEVs Purification

The sEVs were purified from the cell culture supernatant by differential centrifugation according to the procedure previously reported.^[^ ^50^ ^]^ Briefly, fetal bovine serum (FBS) used for sEV extraction was ultracentrifuged at 150 000 × *g* for 12 h at 4 °C before the preparation of the sEV‐free medium. The cells were harvested in the prepared sEV‐free medium for 48 h and the cell culture supernatant was collected. The collected cell culture medium was centrifuged at 800 × *g* for 5 min and 2000 g for 10 min to remove the cell debris, filtered through a 0.22 µm pore filter (Millipore, USA) to remove the large EVs, and then ultracentrifuged twice at 100 000 g for 2 h at 4 °C to pellet the sEVs. As significant deformation of the sEVs were observed over a week of storage (Figure S13, Supporting Information), the sEV pellets were resuspended in 100 µL of PBS and stored at −20 °C for no more than one week prior to use.

### Nanoparticle Tracking Analysis

The size distribution of EVs was measured by setting camera level 16 with an acquisition time of 60 s and a detection threshold setting of 5 at 26 °C based on the NanoSight LM14 system with a 405 nm laser (NanoSight Technology, Malvern, UK). Finally, the data were analyzed using nanoparticle tracking analysis software (NTA version 2.3; Malvern Instruments, Malvern, UK).

### Tip Calibration

Cantilever A was kept and the other five cantilevers of the MLCT probe (Bruker) were knocked out. All the experiments were performed in PBS solution. The fused silica was used to calibrate the probe's deflection sensitivity. Briefly, the force‐indentation curves were obtained on a relative hard sample based on contact mode. Afterward, the contact area between the probe and the sample within the force curve was selected to calculate the reflection sensitivity. The thermal tune mode was used to calibrate the K (elastic coefficient) of the probe. Then the thermal vibration energy of the cantilever was analyzed by a fitting curve. After obtaining deflection sensitivity and K, a complete picture of the standard roughness sample (RS‐15M) was captured using ScanAsyst mode to correct tip radius.

### AFM Imaging

The freshly cleaved mica was cleaned with 3% hydrochloric acid with 96% ethanol for 20 times. Afterward, they were coated for 1 h in a 0.001% poly‐l‐lysine (Solarbio) solution, rinsed with Milli‐Q water (18.2 MΩ.cm), and dried overnight at room temperature. The poly‐l‐lysine modified mica was stored at 4 °C for no more than one month before use. The solution of sEVs was diluted ten times with PBS, filtered with a 0.22 µm filter (Millipore, USA), deposited on the poly‐l‐lysine modified mica for absorbing sEVs and gently rinsed with PBS before imaging. AFM images were acquired in liquid under PeakForce QNM mode on Dimension FastScan AFM (Bruker, Billerica, CA) under the bio model (also called FastScan Bio AFM) using MLCT‐A probes (silicon nitride tip, nominal force constants 0.07 N m^−1^, nominal radius 20 nm, resonant frequency 15–30 kHz, Bruker, US). The imaging force was preset to be 0.5–3 nN. The force‐indentation curves were obtained at an indentation velocity of 250 nm s^−1^ until the surface was reached. The force‐indentation curves of the AFM tip approaching the mica surface were checked before and after each imaging of sEVs to make sure no contaminations from the sEVs adhered on the tip.

### Image Analysis

Images were processed using Nanoscope analysis 1.9 version software. The sizes and shapes of the sEVs were analyzed from the contour of the sEVs obtained in the central axis direction along the largest diameter portion of the profile. Contour correction by deduction of tip radius was performed using the software Nanoscope Analysis (version 1.9) after determination of the tip radius by tip calibration. The contour of the sEV was obtained by smoothing (20 pts, Savitzky Golay Filter) the height profile of the scanned sEV after tip correction. A curvature arc with bilateral symmetry was used to fit the contour from the highest point (*x*, *y*). The radius of the adsorbed sEV (*R*_c_) was estimated from the curvature arc by first derivation as was described in the previous studies.^[^ ^15^, ^51^ ^]^ The height of FIC (*H*_FIC_) was calculated from the FIC. The initial radius *R*_0_ was then calculated assuming that the surface area of the sEV is constant.

### AFM FIC Analysis

The cantilever's response was measured on the sample surface and fitted linearly. The resulting fit was subtracted from the measured response when indenting the vesicles to obtain the FICs. Indentation 0 nm was detected as the first point where the force increased by three times the standard deviation above baseline.^[^ ^52^ ^]^ Stiffness of the EVs was found by fitting a line between 0.05–0.2 *R*_c_. To find the inflection point, FICs were smoothed further and the derivative was taken numerically. Outward tether events were distinguished by the slope after the break event, where tether plateaus had a maximum angle of 0–1°. For the dimensionless fitting in Figure 4a an interpolating function through 13 calculated theoretical pairs of values was created in Mathematica.^[^ ^15^ ^]^ The sum of the squared Euclidian distance between the logarithm of the resulting curve and the logarithm of individual data points was then minimized. Then a set of observed value combinations was randomly drawn and fitted.

### Statistical Analysis

FICs for analyzing stiffness, osmotic pressure, and bending modulus were obtained by correcting baseline and smoothing with 100 pts (Savitzky Golay Filter). Quantitative analysis was conducted by GraphPad Prism software (Version 8.0.1, GraphPad Company, CA) and Minitab Statistical Software (Version 19.1, Minitab Ltd., UK). Origin Pro software (Version 9.7.5.184, Origin‐Lab Company, USA) was used to generate the graphs. Results were presented as mean ± standard deviation (s.d.). The sample size (*N*) for each experiment was indicated in the relevant figure legends. For the statistics of bending modulus, data are presented as mean ± standard error of the mean (s.e.m.) determined by bootstrapping (1000 repetitions). Data were analyzed using the unpaired two‐tailed Student's *t*‐test or one‐way ANOVA followed by a Bonferroni test hoc test, and the statistical significance was displayed as * p < 0.05, ** p < 0.01, *** p < 0.001, **** p < 0.0001, ns not significant and was indicated with ns > 0.05.

## Conflict of Interest

The authors declare no conflict of interest.

## Supporting information

Supporting Information

## Data Availability

Research data are not shared.
