# Supplementary material for: Quantitative Nanomechanical Analysis of Small Extracellular Vesicles for Tumor Malignancy Indication
Source: Adv Sci (Weinh). 2021 Aug 2;8(18):2100825. doi: 10.1002/advs.202100825 (PMC8456224; doi:10.1002/advs.202100825)
Supplement: Supplementary file 1 — Supporting Information [file ADVS-8-2100825-s001.pdf]

© 2021 Wiley-VCH GmbH

ADVANCED SCIENCE
Open Access

Supporting Information

for *Adv. Sci.*, DOI: 10.1002/advs.202100825

Quantitative Nanomechanical Analysis of Small Extracellular Vesicles for Tumor Malignancy Indication

Siyuan Ye, Wenzhe Li, Huayi Wang, Ling Zhu, Chen Wang*, Yanlian Yang**

Supporting Information

Quantitative nanomechanical analysis of small extracellular vesicles for tumor malignancy indication

Siyuan Ye, Wenzhe Li, Huayi Wang, Ling Zhu, Chen Wang*, Yanlian Yang**

S. Ye, Dr. W. Li, Dr. H. Wang, Dr. L. Zhu, Prof. Ch. Wang, Prof. Y. Yang
CAS Key Laboratory of Standardization and Measurement for Nanotechnology
CAS Key Laboratory of Biological Effects of Nanomaterials and Nanosafety
CAS Center for Excellence in Nanoscience
National Center for Nanoscience and Technology
Beijing 100190, P. R. China
E-mail: zhul@nanoctr.cn; wangch@nanoctr.cn; yangyl@nanoctr.cn

S. Ye, Dr. H. Wang, Dr. L. Zhu, Prof. Ch. Wang, Prof. Y. Yang
University of Chinese Academy of Sciences
Beijing 100049, P. R. China

S. Ye
Department of Chemistry
Tsinghua University
Beijing, 100084, P. R. China

Dr. W. Li
State Key Laboratory of Natural and Biomimetic Drugs
School of Pharmaceutical Sciences
Peking University
Beijing 100871, P. R. China

Dr. H. Wang
Chinese Institute for Brain Research (CIBR)
Beijing 102206, P. R. China

This file includes:

Supplementary Figures

Figure S1. AFM topographic images of the same sEV under the indentation forces varying from 0.5 nN - 3 nN.

Figure S2. Determination of the radius of the adsorbed sEVs.

Figure S3. Force-indentation curves obtained with tips ($R_t \approx 20$ nm) on the sEV from MCF-10A cell lines.

Figure S4. Rupture of the sEV by nanoindentation.

Figure S5. The choose of Canham-Helfrich model to extract individual nanomechanical properties of the sEVs from the FICs.

Figure S6. Estimation of the bending modulus of the sEVs released from MCF-7, SK-BR-3, MDA-MB-468, and MDA-MB-231.

Figure S7. Outward tether force (F_t) calculation.

Figure S8. The bending modulus of different sEV subpopulations. The bending modulus of each sEV subpopulation was calculated using AFM nano-indentation based on Canham-Helfrich theory.

Figure S9. Receiver operating characteristic (ROC) analysis showing the discriminative efficacy of the bending modulus, osmotic pressure, and stiffness of the sEVs in distinguishing high-malignant and low-malignant sEVs.

Figure S10. Receiver operating characteristic (ROC) analysis showing the discriminative efficacy of the bending modulus of the sEVs.

Figure S11. Receiver operating characteristic (ROC) analysis showing the discriminative efficacy of the osmotic pressure of the sEVs.

Figure S12. Receiver operating characteristic (ROC) analysis showing the discriminative efficacy of the stiffness of the sEVs.

Figure S13. AFM topographic images of small extracellular vesicles (sEVs) after storage at -20°C for one week.

Supplementary Tables

Table S1. Stiffness of the sEVs from different cell lines.

Table S2. Detailed values obtained from the ROC analysis of the osmotic pressure, bending modulus, and stiffness of the sEVs in distinguishing sEVs derived from different cell lines between each pair.

Table S3. Detailed values of bending modulus obtained from the ROC analysis in paired sEV subgroups with different size range in distinguishing sEVs derived from different cell lines.

Table S4. Detailed values of osmotic pressure obtained from the ROC analysis in paired sEV subgroups with different size range in distinguishing sEVs derived from different cell lines.

Table S5. Detailed values of stiffness obtained from the ROC analysis in paired sEV subgroups with different size range in distinguishing sEVs derived from different cell lines

Supplementary Figures and Tables

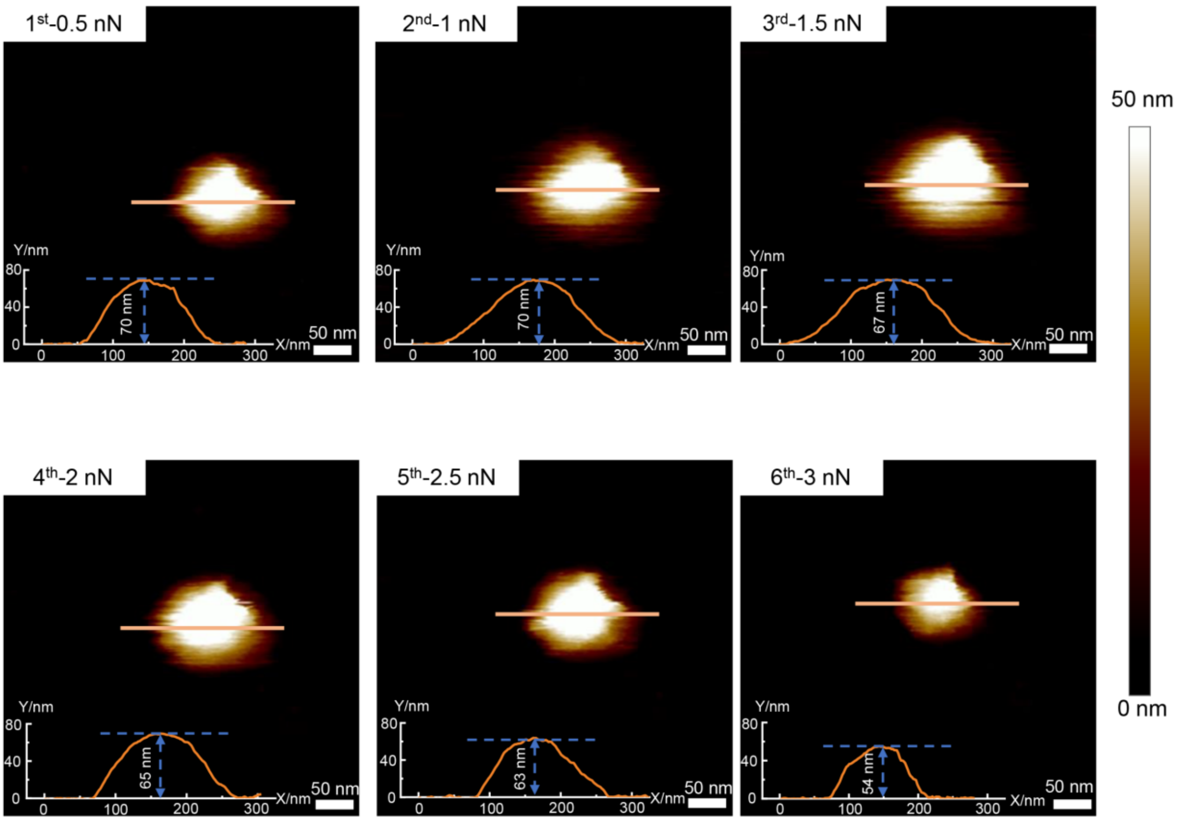

Figure S1. AFM topographic images of the same sEV under the indentation forces varying from 0.5 nN - 3 nN. Inset: the tip-corrected contour of the sEV through the maximum height of the same sEV (along the orange solid line). The height of the same sEV was slightly different under different forces.

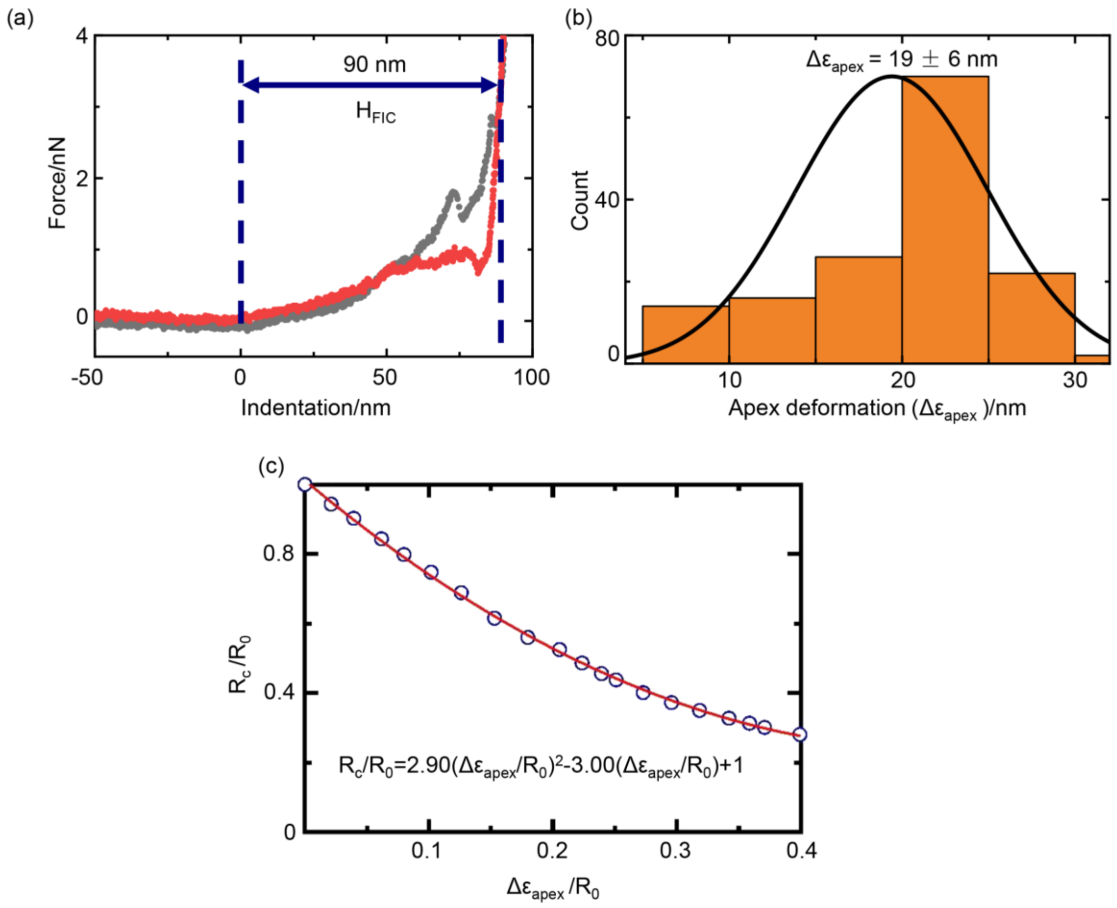

Figure S2. Determination of the radius of the adsorbed sEVs. (a) Force-indentation curve (FIC) of the sEV. The height of the sEV without deformation (H_{FIC} = 90 nm) can be determined by measuring the distance between the contact point at zero force and the maximum indentation. (b) Histogram of the apex deformation of the sEVs at the imaging force of 1 nN. The deformation of the apex ($\Delta\varepsilon_{apex}$) of each sEV is calculated by subtracting H_{TI} from H_{FIC}. The mean deformation of the apex of sEVs was determined to be 19 ± 6 nm (st.d., N = 150 sEVs). (c) The relationship between $\Delta\varepsilon_{apex}$ and R_c. Both $\Delta\varepsilon_{apex}$ and R_c are normalized by dividing with the initial radius of the sEV (R_0) before being adsorbed on the substrate. R_0 was estimated from the surface area of the sEVs approximated from the AFM topographic images, assuming that the surface area of the sEVs was constant after adhering to the substrate. The blue circles are the data obtained from an individual sEV under the setpoint force varying from 0.5 to 3 nN. We therefore performed a simulation of the data points and determined the relationship between $\Delta\varepsilon_{apex}$ and R_c as $R_c/R_0 = 2.90\ (\Delta\varepsilon_{apex}/R_0)^2-3.00\ (\Delta\varepsilon_{apex}/R_0)+1$ (ref solid line) which allows us to use 3 times the deformation of the apex to simplify the mathematical to determine R_c of the sEVs from their $\Delta\varepsilon_{apex}$.

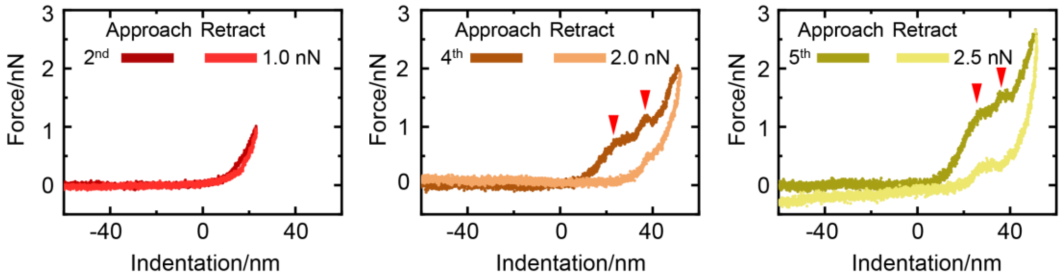

Figure S3. Force-indentation curves obtained with tips ($R_t \approx 20$ nm) on the sEV from MCF-10A cell lines. FICs obtained from the 2nd (1 nN), 4th (2.0 nN), and 5th (2.5 nN) indentations in the sequential indentation experiment in Figure. 3a. The darker colors represented the approaching curves and the lighter colors represented the retracting curves. The 2nd FIC was similar to the 1st one that showed an initial linear elastic response under 1 nN. The 4th and 5th FICs were similar to the 3rd one that suggested twice penetration of the sEV membrane. The breaks indicating membrane penetration are marked with red triangles.

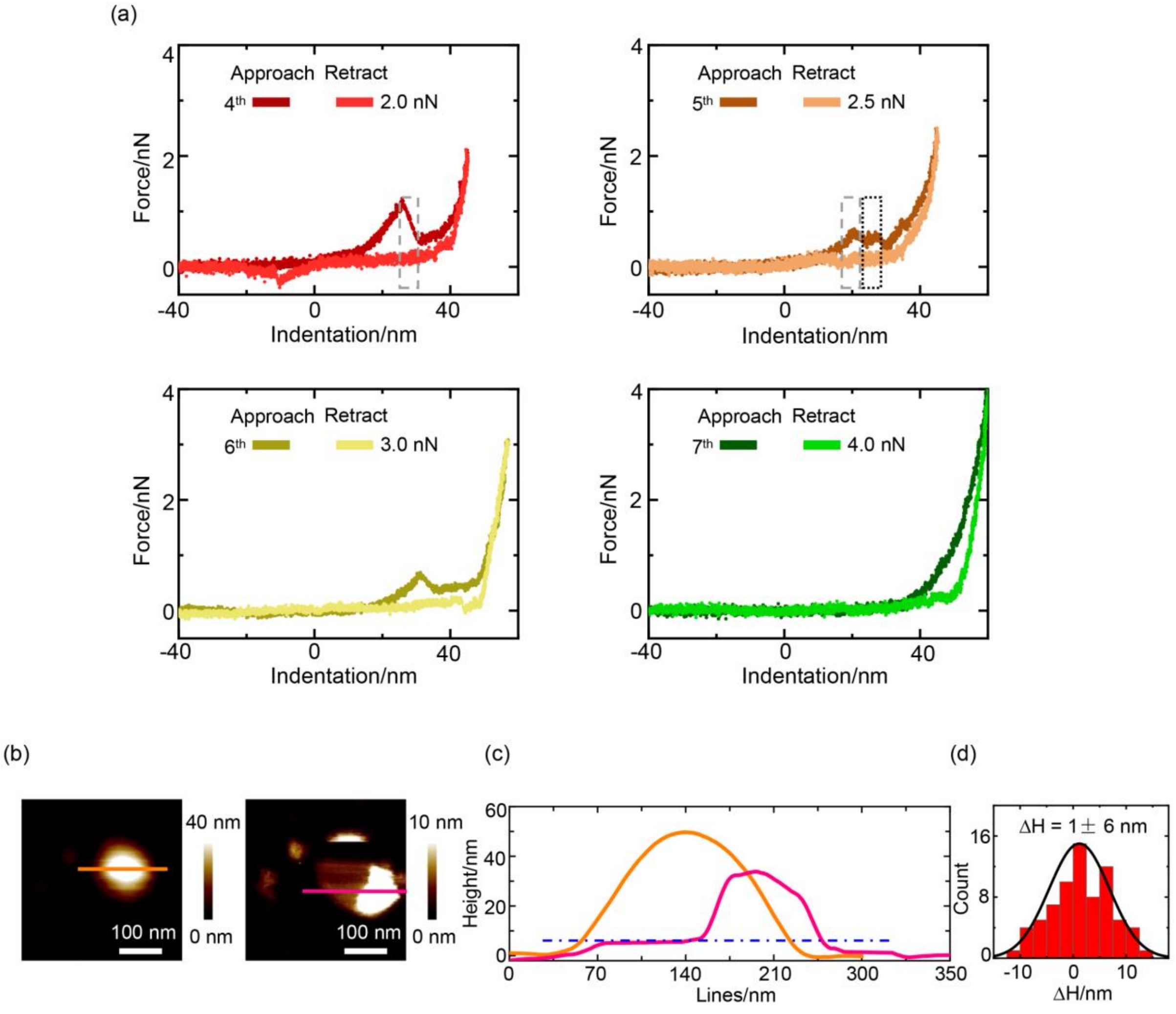

Figure S4. Rupture of the sEV by nanoindentation. (a) Representative FICs showing the sequential indentations on the same sEV with increasing forces. The dark colors represent the approach curve and the light colors represent the retract curve. The large irreversible break in the 4th indentation (2.0 nN) suggested membrane penetration and strong deformation of the sEV. The size of such fracture (~ 4nm) corresponded well to the thickness of the phospholipid bilayer[1] (represented by a gray dashed box). In the subsequent indentation process (5th, 2.5 nN), the sEV presented continuous breaks (~ 3.2 nm, represented by a dashed box in gray and dot box in black, respectively.) The height of these fractures was in accordance with the thickness of the two phospholipid bilayers. After further indentations (6th, 3.0 nN, 7th, 4.0 nN), the slope of the linear force response stage significantly increased (superlinear response), suggesting possible rupture of the sEV. (b) AFM topographic images of showing different geometry of the same sEV before and after six indentations. (c) Height profiles through the maximum height of the same sEV along the colored lines in (b) before the 1st indentation (orange) and after the 6th indentation (pink). (The blue dashed line indicated the thickness of

the phospholipid bilayer). (d) Histogram showed the height differences of the sEVs before and after sequential indentations. 72 MCF-10A-derived sEVs were analyzed ($\Delta H = 1 \pm 6$ nm, st.d.).

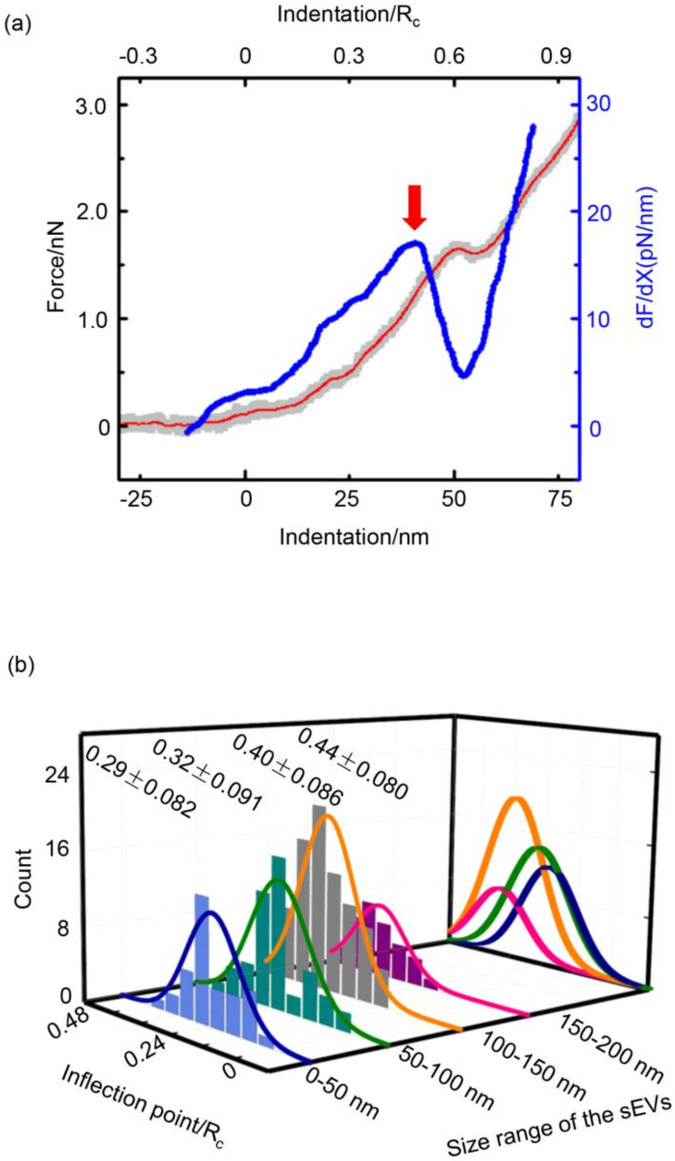

Figure S5. The choose of Canham-Helfrich model to extract individual nanomechanical properties of the sEVs from the FICs. (a) Determination of the infection point where the inward tether force formed. The approach curve with the typical plateau and breaks that represented the formation of inward tether force and membrane penetration was analyzed. The experimental data points (grey) in a typical FIC were smoothed (red curve) and the second-order derivative of the FIC was calculated (blue curve). The first peak (marked with red arrow) in the derivative curve was determined to be the inflection point. (b) Thin shell elastic model and Canham-Helfrich model are the two common models for the nanomechanical analysis of the vesicles. In thin shell elastic theory, the vesicle is viewed as a double-layer elastic thin shell with uniform liquid enclosed, regardless of the fluidity and spontaneous curvature of the vesicle membrane,[2] while in Canham-Helfrich theory, the shape of the vesicle is determined by the minimum free energy considering the spontaneous

curvature of the membrane.[3-5] The histogram shows that the inflection points of the sEVs with different sizes all fell into the range of 0.3 ~ 0.45 R_c, much deeper than the one predicted from the thin shell elastic model (0.05 ~ 0.07 R_c),[6] suggesting that the mechanical behavior of sEVs was mainly determined by the fluid membrane. Solid lines represent Gaussian fits. The sEVs were derived from MCF-10A cells. Displayed is the mean ± st.d..

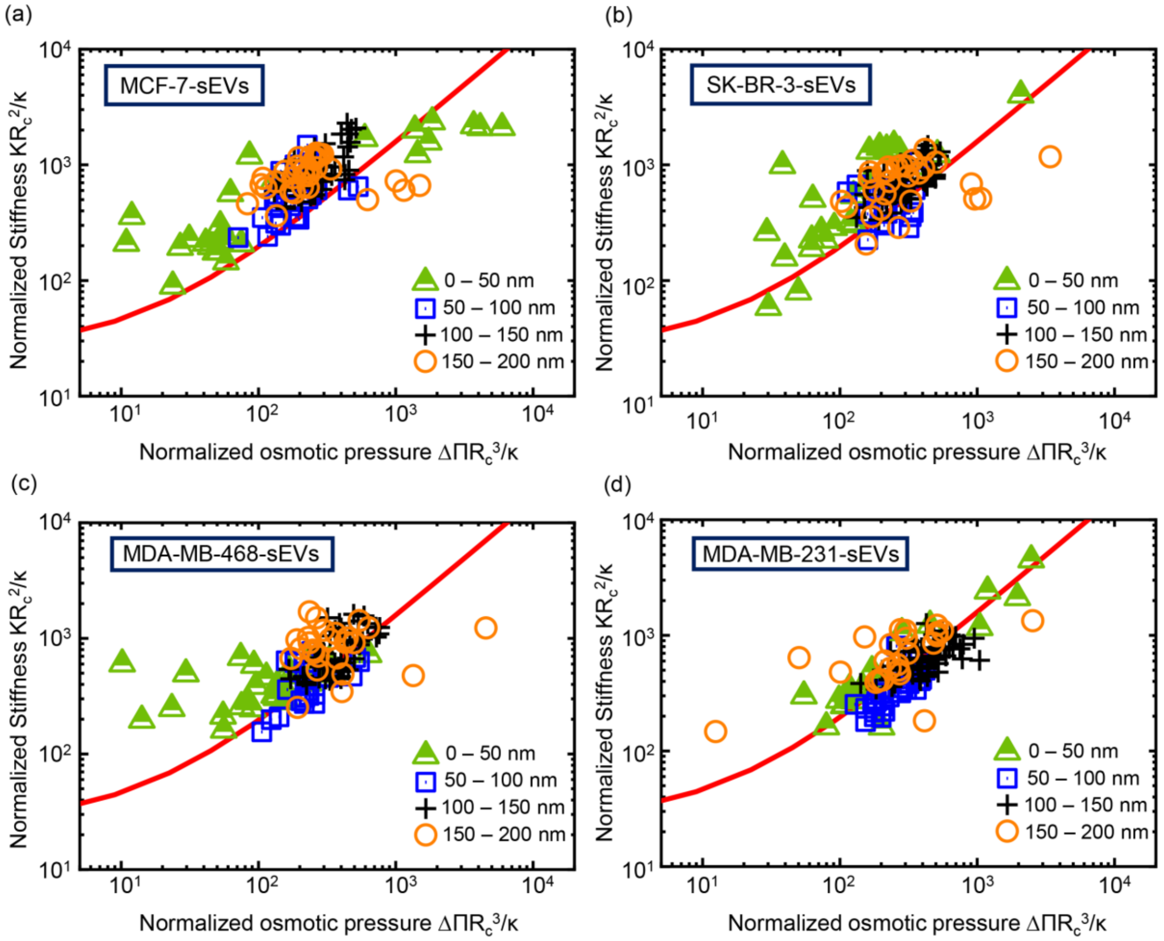

Figure S6. Estimation of the bending modulus of the sEVs released from a) MCF-7, b) SK-BR-3, c) MDA-MB-468, and d) MDA-MB-231. The plot showing the relationship between the normalized osmotic pressure and the normalized stiffness. Theoretical prediction (solid red curve) is based on a recent model describing the mechanical behavior of the small lipid vesicles according to Canham-Helfrich theory.[3-5] Markers represented the experimental data from the sEVs. The bending modulus was determined by minimizing the sum of the squared Euclidian distance between the logarithm of the theoretically predicted curve and the experimental values (described in detail in the main text). The numbers of sEVs included for analysis are (in the order of MCF-7, SK-BR-3, MDA-MB-468 and MDA-MB-231): 0 - 50 nm: N = 30, 26, 24, 26, 23; 50 - 100 nm: N = 39, 28, 24, 22, 31; 100 - 150 nm: N = 57, 46, 49, 52, 57; 150 - 200 nm: N = 23, 33, 28, 25, 24.

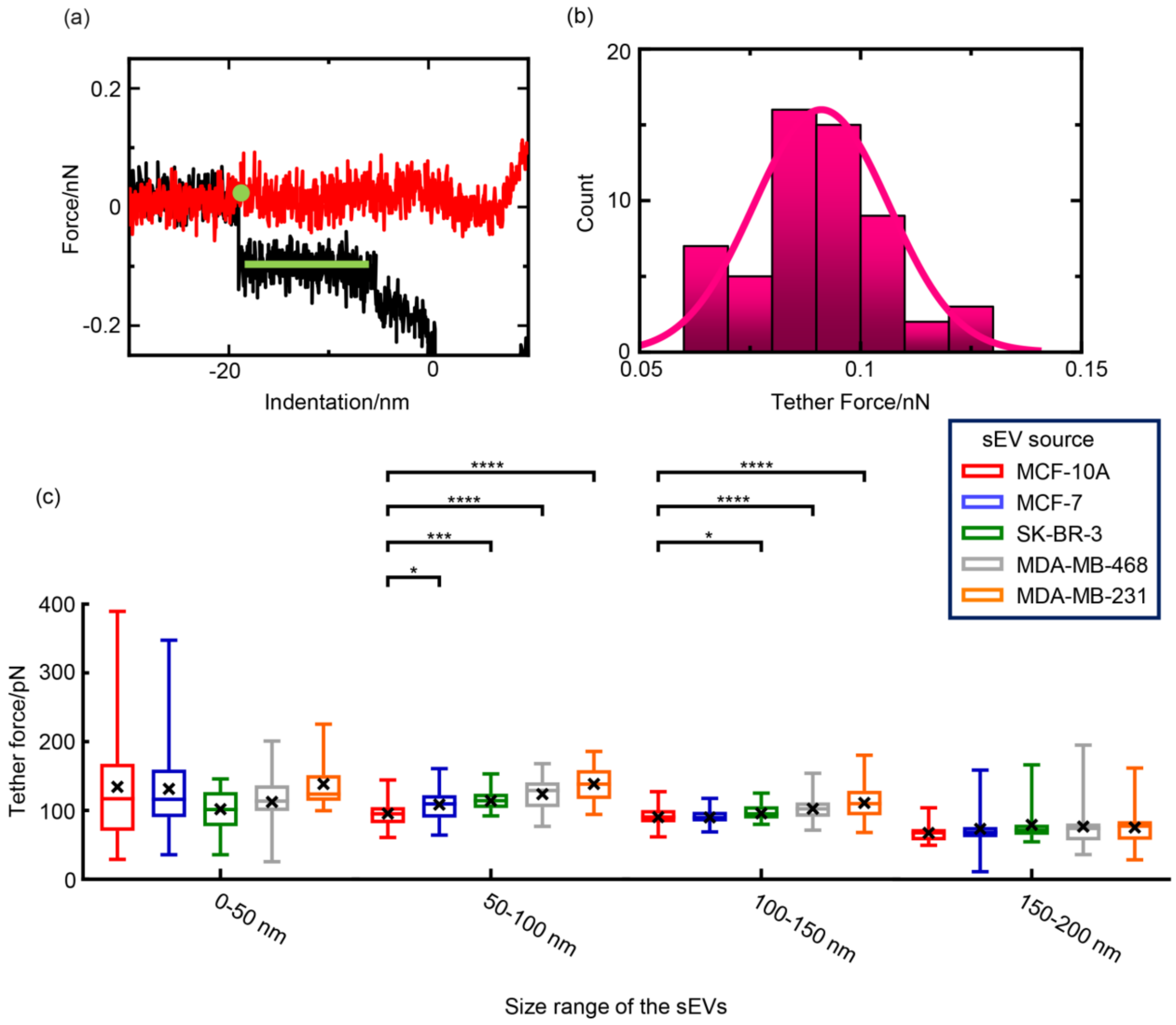

Figure S7. Outward tether force (F_t) calculation. (a) Force measurement from a typical FIC (approach in red and retrace in black). Green lines indicate the fitted regions where the slope is ~ 0. The outward tether events are recorded when extruding a membrane tether from the sEV membrane with the molecular contents of sEV at its tip. (b) Histogram of the F_t was measured from the sEVs at the size range of 100 - 150 nm (N = 57). The sEVs were released from MCF-10A cells. (c) The outward tether force of the sEVs with different sizes and from different cell sources. The box limits indicate upper and lower quartiles and whiskers indicate 1.5x interquartile range. The solid lines in the box plots represent the median value and the crosses indicate the mean. The numbers of sEVs included for analysis are (in the order of MCF-10A, MCF-7, SK-BR-3, MDA-MB-468 and MDA-MB-231): 0 - 50 nm: N = 30, 26, 24, 26, 23; 50 - 100 nm: N = 39, 28, 24, 22, 31; 100 - 150 nm: N = 57, 46, 49, 52, 57; 150 - 200 nm: N = 23, 33, 28, 25, 24. (*$p < 0.05$, ***$p < 0.001$, ****$p < 0.0001$, Two-tailed t-test).

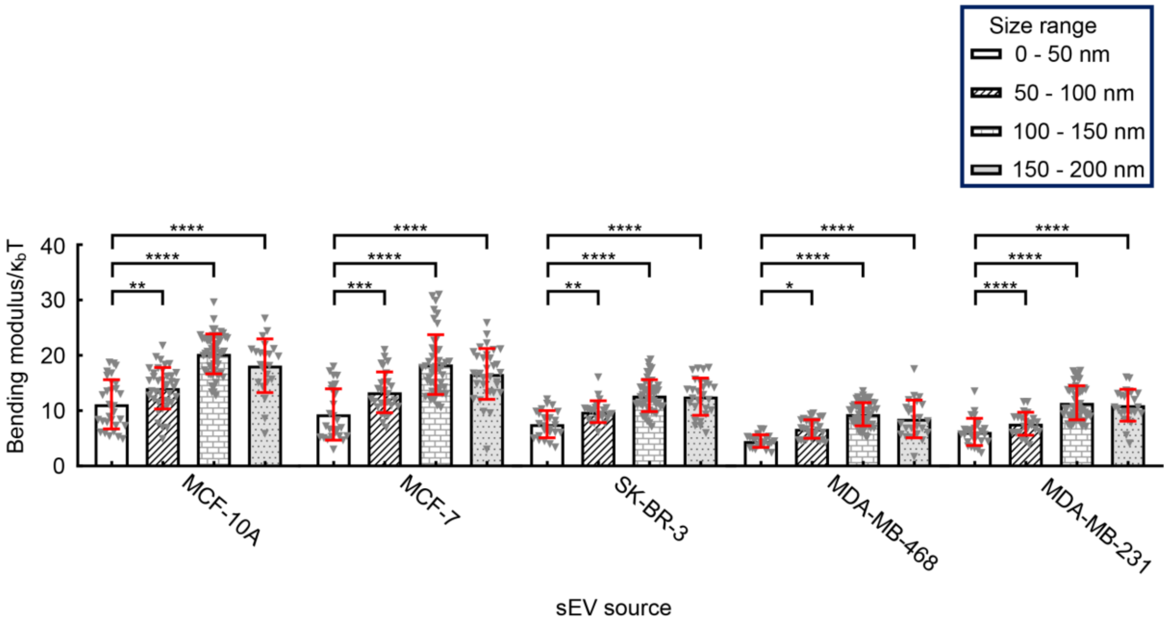

Figure S8. The bending modulus of different sEV subpopulations. The bending modulus of each sEV subpopulation was calculated using AFM nano-indentation based on Canham-Helfrich theory. Histogram bars indicate means, and error bars indicate standard deviation (st.d.). The data points represent the estimated bending modulus of each sEV. The number of sEVs included for the analysis are (in the order of MCF-10A, MCF-7, SK-BR-3, MDA-MB-468 and MDA-MB-231): 0 - 50 nm: N = 30, 26, 24, 26, 23; 50 - 100 nm: N = 39, 28, 24, 22, 31; 100 - 150 nm: N = 57, 46, 49, 52, 57; 150 - 200 nm: N = 23, 33, 28, 25, 24. Data are represented as mean ± st.d.. (*$p < 0.05$, **$p < 0.01$, ***$p < 0.001$, ****$p < 0.0001$, Two-tailed t-test).

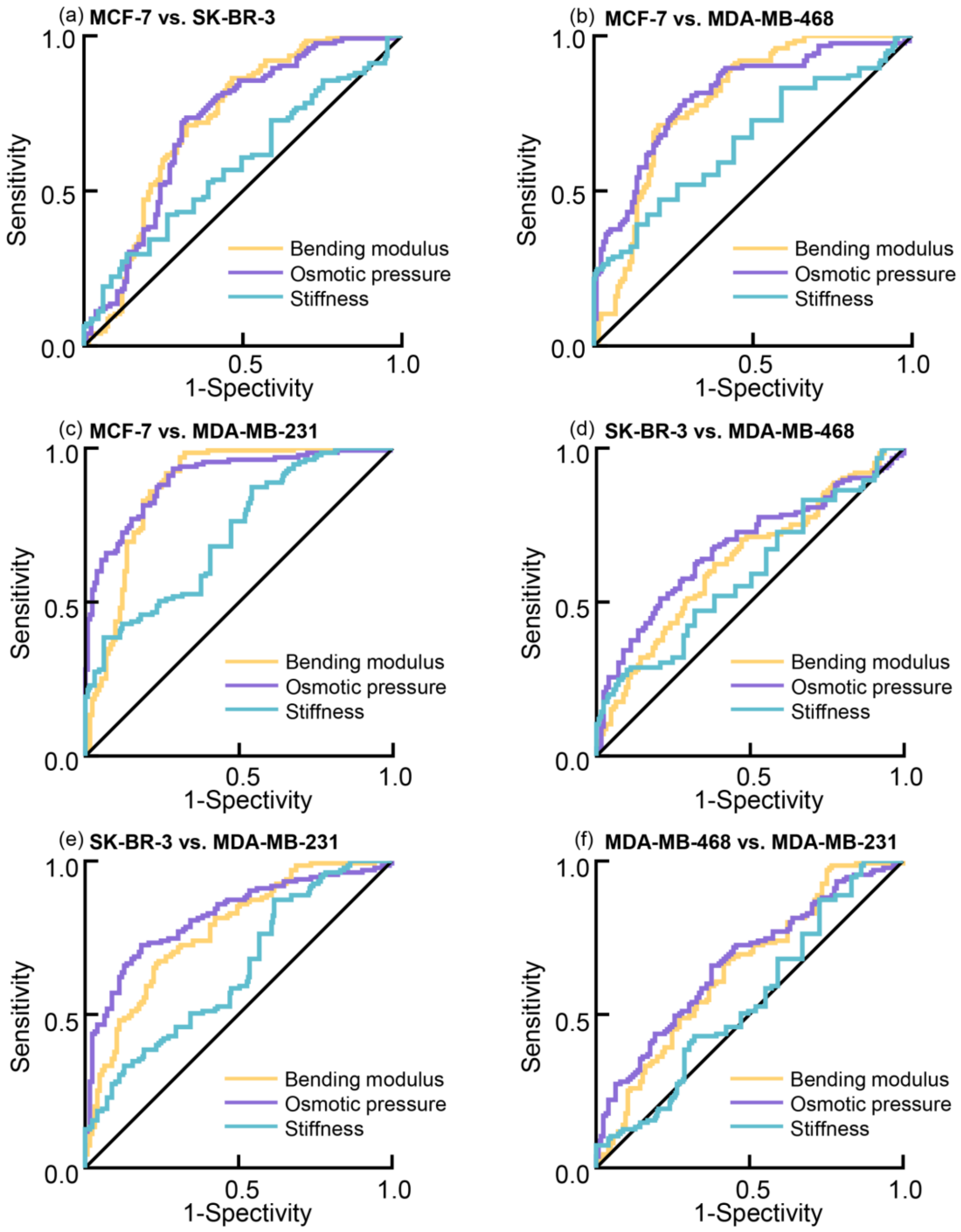

Figure S9. Receiver operating characteristic (ROC) analysis showing the discriminative efficacy of the bending modulus, osmotic pressure, and stiffness of the sEVs in distinguishing high-malignant and low-malignant sEVs. ROC curve of the bending modulus (yellow line), the osmotic pressure (purple line), and the stiffness (blue line) in distinguishing sEVs derived from a) MCF-7 (N = 133) and SK-BR-3 (N = 125), b) MCF-7 (N = 133) and MDA-MB-468 (N = 125), c) MCF-7 (N = 133) and MDA-MB-231 (N = 135), d) SK-BR-3 (N = 125) and MDA-MB-468 (N = 125), e) SK-BR-3 (N = 125) and MDA-MB-231 (N = 135), f) MDA-MB-468 (N = 125) and MDA-MB-231 (N = 135). The area under the ROC curve (AUC) of different group from (a) to (f): 0.7238, 0.7160, 0.5830; 0.7914, 0.8077, 0.6610; 0.8730, 0.8997, 0.7190; 0.6307, 0.6757, 0.5905; 0.7730, 0.8184, 0.6399; 0.6461, 0.6683,

0.5435 (bending modulus, osmotic pressure, and stiffness, respectively). The detailed data about st.d. error, 95% confidence interval, P value and Cut off value were displayed in the Table S2.

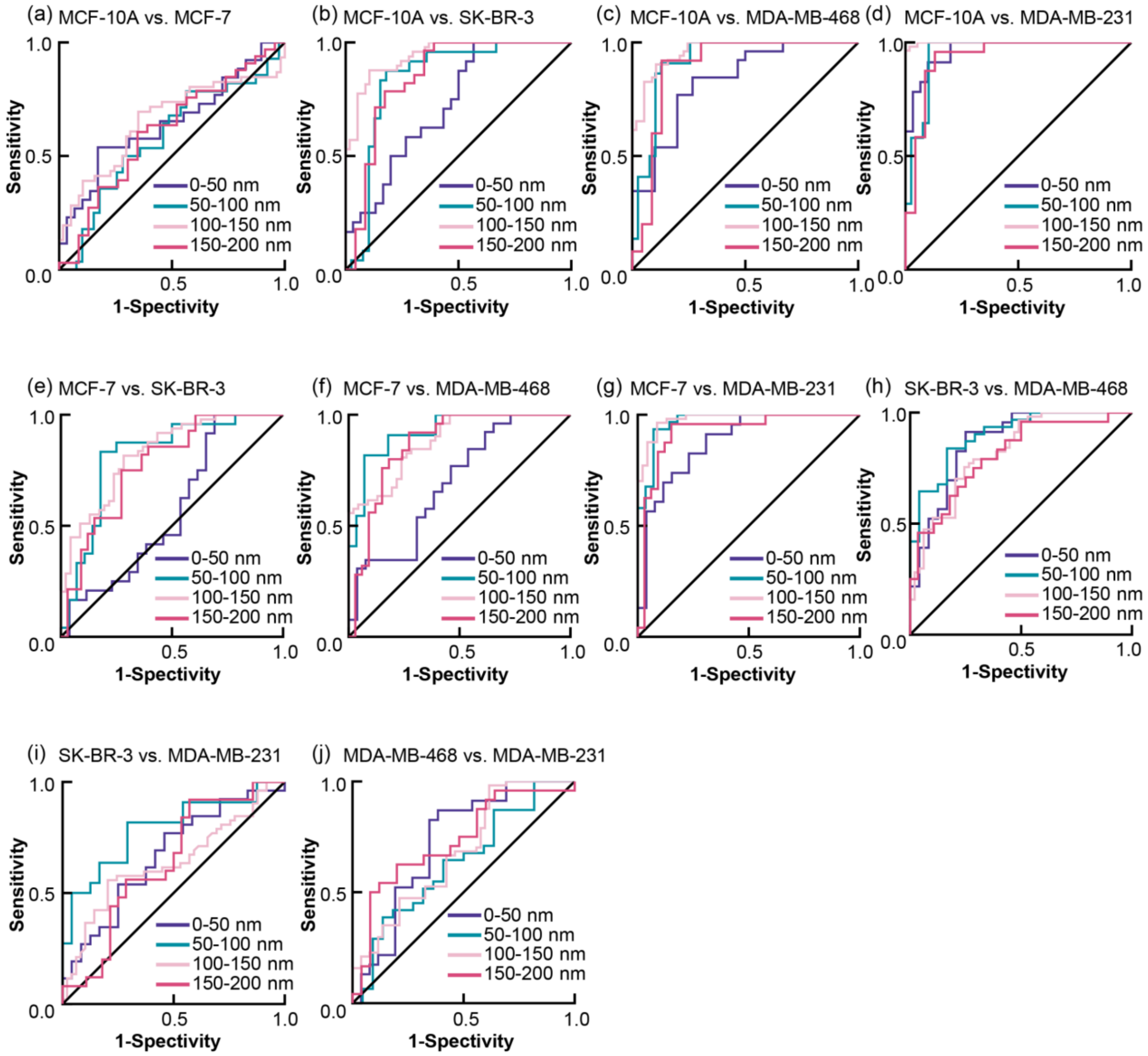

Figure S10. Receiver operating characteristic (ROC) analysis showing the discriminative efficacy of the bending modulus of the sEVs. ROC curve of the sEVs range from 0-50 nm (purple line), 50-100 nm (blue line), 100-150 nm (pink line) and 150-200 nm (dark pink line) in distinguishing sEVs derived from a) MCF-10A and MCF-7, b) MCF-10A and SK-BR-3, c) MCF-10A and MDA-MB-468, d) MCF-10A and MDA-MB-231, e) MCF-7 and SK-BR-3, f) MCF-7 and MDA-MB-468, g) MCF-7 and MDA-MB-231, h) SK-BR-3 and MDA-MB-468, i) SK-BR-3 and MDA-MB-231, j) MDA-MB-468 and MDA-MB-231. (in the order of MCF-10A, MCF-7, SK-BR-3, MDA-MB-468 and MDA-MB-231: 0 - 50 nm: N = 30, 26, 24, 26, 23; 50 - 100 nm: N = 39, 28, 24, 22, 31; 100 - 150 nm: N = 57, 46, 49, 52, 57; 150 - 200 nm: N = 23, 33, 28, 25, 24). The detailed data about the AUC, st.d. error, 95% confidence interval, P value and Cut off value were displayed in the Table S3.

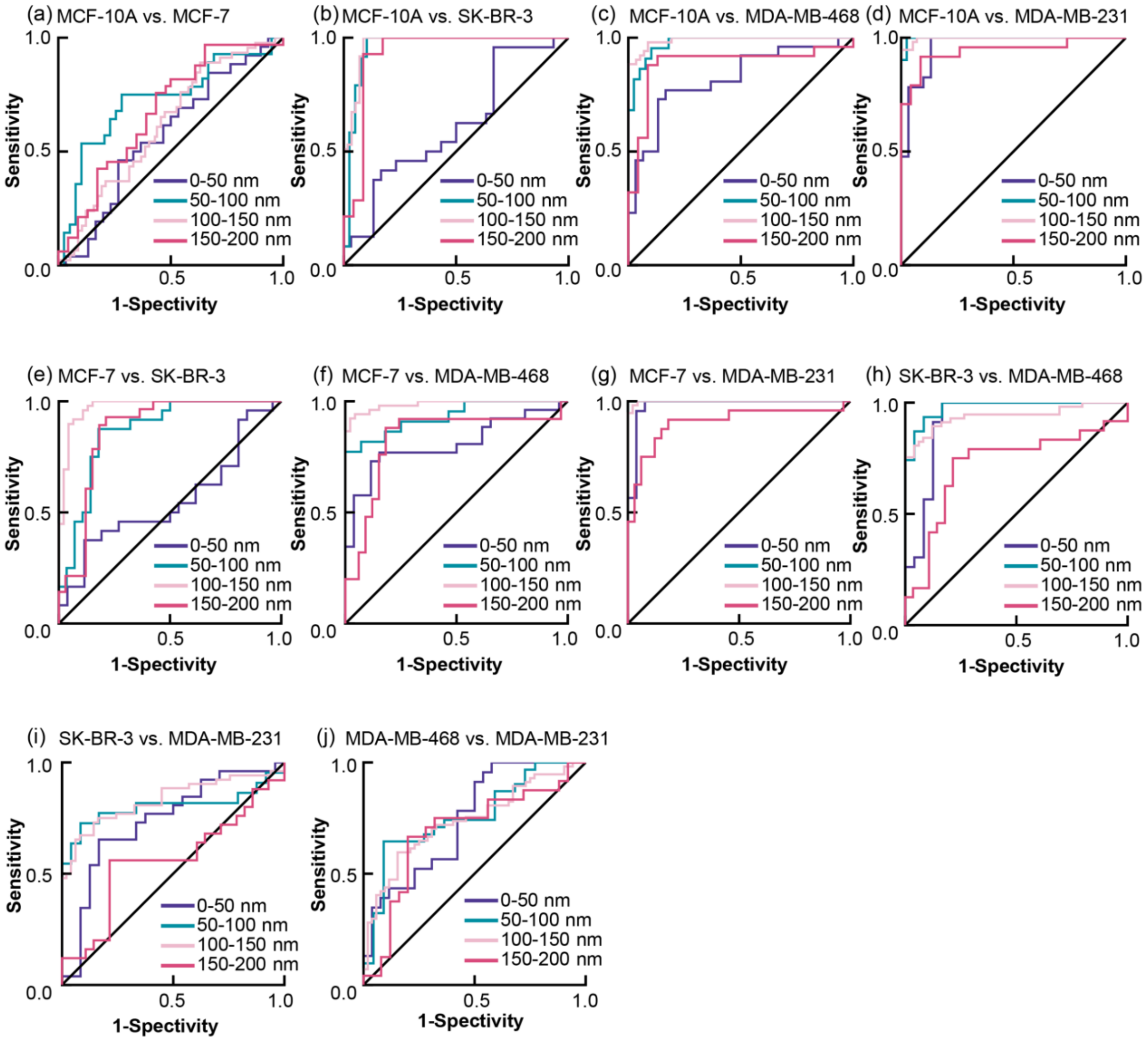

Figure S11. Receiver operating characteristic (ROC) analysis showing the discriminative efficacy of the osmotic pressure of the sEVs. ROC curve of the sEVs range from 0-50 nm (purple line), 50-100 nm (blue line), 100-150 nm (pink line) and 150-200 nm (dark pink line) in distinguishing sEVs derived from a) MCF-10A and MCF-7, b) MCF-10A and SK-BR-3, c) MCF-10A and MDA-MB-468, d) MCF-10A and MDA-MB-231, e) MCF-7 and SK-BR-3, f) MCF-7 and MDA-MB-468, g) MCF-7 and MDA-MB-231, h) SK-BR-3 and MDA-MB-468, i) SK-BR-3 and MDA-MB-231, j) MDA-MB-468 and MDA-MB-231. (in the order of MCF-10A, MCF-7, SK-BR-3, MDA-MB-468 and MDA-MB-231: 0 - 50 nm: N = 30, 26, 24, 26, 23; 50 - 100 nm: N = 39, 28, 24, 22, 31; 100 - 150 nm: N = 57, 46, 49, 52, 57; 150 - 200 nm: N = 23, 33, 28, 25, 24). The detailed data about the AUC, st.d. error, 95% confidence interval, P value and Cut off value were displayed in the Table S4.

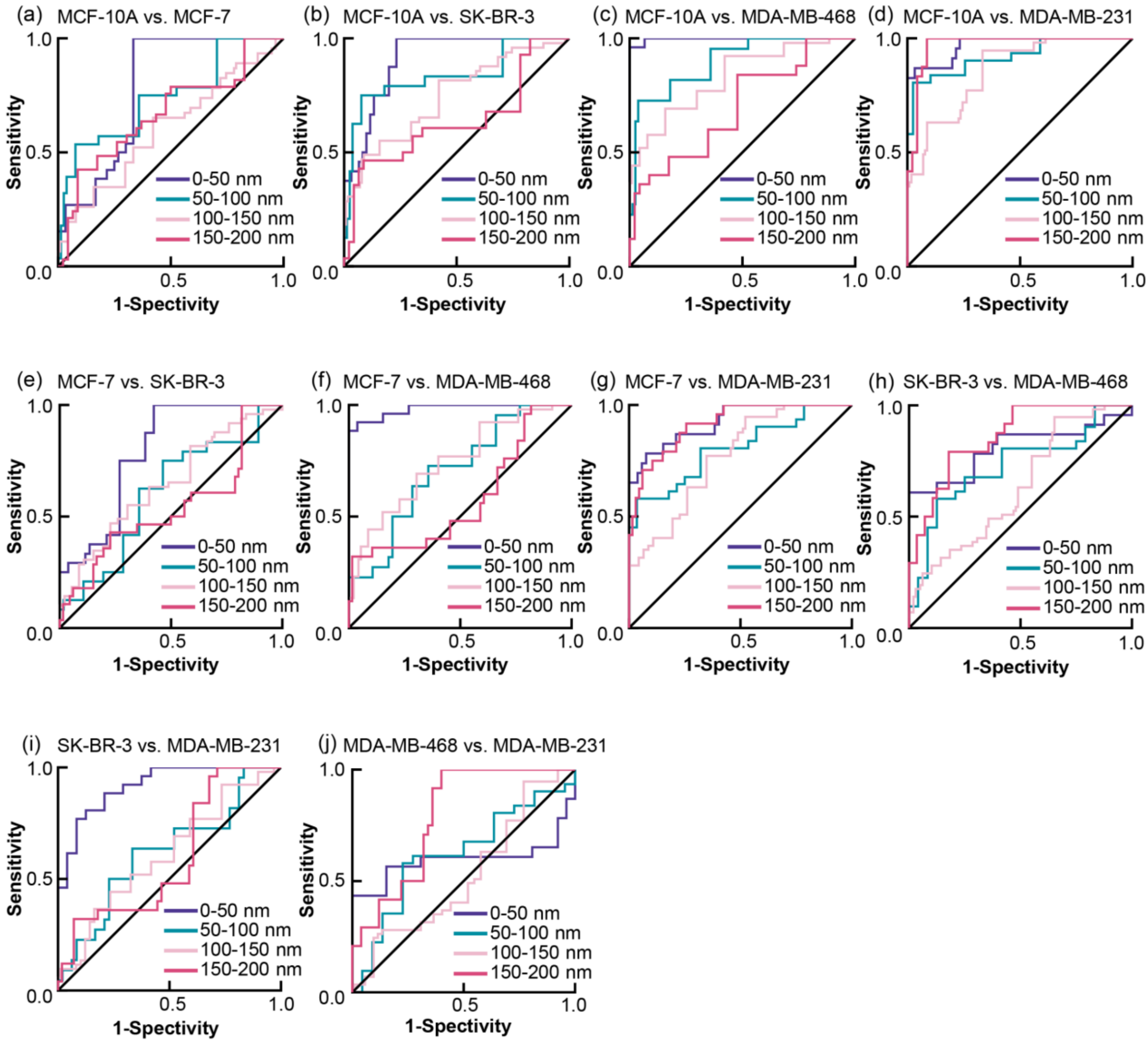

Figure S12. Receiver operating characteristic (ROC) analysis showing the discriminative efficacy of the stiffness of the sEVs. ROC curve of the sEVs range from 0-50 nm (purple line), 50-100 nm (blue line), 100-150 nm (pink line) and 150-200 nm (dark pink line) in distinguishing sEVs derived from a) MCF-10A and MCF-7, b) MCF-10A and SK-BR-3, c) MCF-10A and MDA-MB-468, d) MCF-10A and MDA-MB-231, e) MCF-7 and SK-BR-3, f) MCF-7 and MDA-MB-468, g) MCF-7 and MDA-MB-231, h) SK-BR-3 and MDA-MB-468, i) SK-BR-3 and MDA-MB-231, j) MDA-MB-468 and MDA-MB-231. (in the order of MCF-10A, MCF-7, SK-BR-3, MDA-MB-468 and MDA-MB-231: 0 - 50 nm: N = 30, 26, 24, 26, 23; 50 - 100 nm: N = 39, 28, 24, 22, 31; 100 - 150 nm: N = 57, 46, 49, 52, 57; 150 - 200 nm: N = 23, 33, 28, 25, 24). The detailed data about the AUC, st.d. error, 95% confidence interval, P value and Cut off value were displayed in the Table S5.

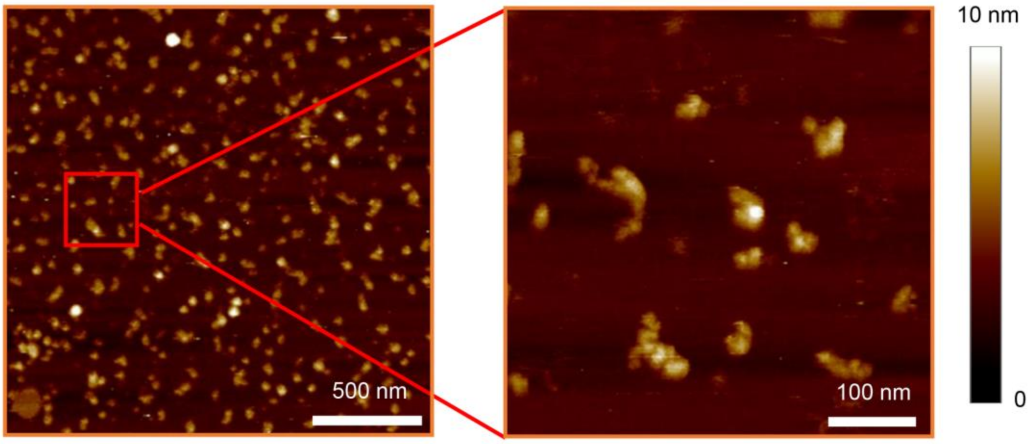

Figure S13. AFM topographic images of small extracellular vesicles (sEVs) after storage at -20°C for one week. The sEVs were derived from MCF-10A cell line. The sEVs were adsorbed on the poly-L-lysine-coated mica and were imaged in liquid under PeakForce QNM mode. Great changes in the shape and height of EVs can be observed after a long time (over one week) storage.

Table S1. Stiffness of the sEVs from different cell lines.

Size range of the sEVs (nm)	sEV source	Stiffness/K ($N\ m^{-1}$ ± st.d.)	Number of sEV/N
0 - 50	MCF-10A	0.014 ± 0.0041	30
	MCF-7	0.018 ± 0.0035	26
	SK-BR-3	0.022 ± 0.0029	24
	MDA-MB-468	0.028 ± 0.0031	26
	MDA-MB-231	0.033 ± 0.011	23
50 - 100	MCF-10A	0.011 ± 0.0021	39
	MCF-7	0.014 ± 0.0037	28
	SK-BR-3	0.016 ± 0.0047	24
	MDA-MB-468	0.018 ± 0.0057	22
	MDA-MB-231	0.020 ± 0.0060	31
100 - 150	MCF-10A	0.010 ± 0.0018	57
	MCF-7	0.011 ± 0.0026	46
	SK-BR-3	0.013 ± 0.0037	49
	MDA-MB-468	0.014 ± 0.0044	52
	MDA-MB-231	0.015 ± 0.0046	57
150 - 200	MCF-10A	0.0066 ± 0.0017	23
	MCF-7	0.0074 ± 0.0016	33
	SK-BR-3	0.0076 ± 0.0017	28
	MDA-MB-468	0.0082 ± 0.0018	25
	MDA-MB-231	0.010 ± 0.0018	24

Table S2. Detailed values obtained from the ROC analysis of the osmotic pressure, bending modulus, and stiffness of the sEVs in distinguishing sEVs derived from different cell lines between each pair.

sEV source	Mechanical property	Area	St.d error	95% confidence interval	P value	Cut off value
MCF-10A vs. MCF-7	Bending modulus	0.5856	0.03396	0.5190 to 0.6522	0.0131	< 15.16
	Osmotic pressure	0.5631	0.03423	0.4960 to 0.6301	0.0676	> 0.06897
	Stiffness	0.5737	0.03497	0.5052 to 0.6423	0.0325	> 0.01388
MCF-10A vs. SK-BR-3	Bending modulus	0.7915	0.02765	0.7373 to 0.8456	<0.0001	< 14.34
	Osmotic pressure	0.7824	0.02808	0.7274 to 0.8374	<0.0001	> 0.06773
	Stiffness	0.6522	0.03462	0.5843 to 0.7200	<0.0001	> 0.01388
MCF-10A vs. MDA-MB-468	Bending modulus	0.8453	0.02358	0.7990 to 0.8915	<0.0001	< 14.28
	Osmotic pressure	0.8496	0.02413	0.8023 to 0.8969	<0.0001	> 0.1062
	Stiffness	0.7266	0.03199	0.6639 to 0.7893	<0.0001	> 0.01450
MCF-10A vs. MDA-MB-231	Bending modulus	0.9099	0.01775	0.8751 to 0.9447	<0.0001	< 12.79
	Osmotic pressure	0.9210	0.01655	0.8886 to 0.9534	<0.0001	> 0.1075
	Stiffness	0.7931	0.02608	0.7420 to 0.8442	<0.0001	> 0.01205
MCF-7 vs. SK-BR-3	Bending modulus	0.7238	0.03205	0.6610 to 0.7867	0.0001	< 14.80
	Osmotic pressure	0.7160	0.03222	0.6528 to 0.7791	<0.0001	> 0.07690
	Stiffness	0.5830	0.03556	0.5133 to 0.6527	0.0212	> 0.01808
MCF-7 vs. MDA-MB-468	Bending modulus	0.7914	0.02846	0.7356 to 0.8472	<0.0001	< 10.81
	Osmotic pressure	0.8077	0.02712	0.7546 to 0.8608	<0.0001	> 0.08577
	Stiffness	0.6610	0.03392	0.5945 to 0.7275	<0.0001	> 0.01584
MCF-7 vs. MDA-MB-231	Bending modulus	0.8730	0.02281	0.8283 to 0.9177	<0.0001	< 12.56
	Osmotic pressure	0.8997	0.01866	0.8632 to 0.9363	<0.0001	> 0.08602
	Stiffness	0.7190	0.03051	0.6592 to 0.7788	<0.0001	> 0.01010
SK-BR-3 vs. MDA-MB-468	Bending modulus	0.6307	0.03512	0.5619 to 0.6995	0.0004	< 10.03
	Osmotic pressure	0.6757	0.03426	0.6086 to 0.7429	<0.0001	> 0.1421
	Stiffness	0.5905	0.03588	0.5201 to 0.6608	0.0134	> 0.02173
SK-BR-3 vs. MDA-MB-231	Bending modulus	0.7730	0.02864	0.7168 to 0.8291	<0.0001	< 8.639
	Osmotic pressure	0.8184	0.02637	0.7667 to 0.8701	<0.0001	> 0.1853
	Stiffness	0.6399	0.03404	0.5731 to 0.7066	<0.0001	> 0.01010
MDA-MB-468 vs. MDA-MB-231	Bending modulus	0.6461	0.03411	0.5793 to 0.7129	<0.0001	< 8.640
	Osmotic pressure	0.6683	0.03320	0.6032 to 0.7333	<0.0001	> 0.2249
	Stiffness	0.5435	0.03602	0.4729 to 0.6141	0.2260	> 0.01010

Table S3. Detailed values of bending modulus obtained from the ROC analysis in paired sEV subgroups with different size range in distinguishing sEVs derived from different cell lines.

sEV source	Size range of the sEVs	Area	St.d error	95% confidence interval	P value	Cut off value
MCF-10A vs. MCF-7	0- 50 nm	0.6459	0.07583	0.4973 to 0.7945	0.0637	< 7.326
	50-100 nm	0.5815	0.07230	0.4398 to 0.7232	0.2579	< 15.29
	100-150 nm	0.6560	0.05696	0.5444 to 0.7676	0.0067	< 19.18
	150-200 nm	0.6087	0.07776	0.4563 to 0.7611	0.1694	< 17.74
MCF-10A vs. SK-BR-3	0- 50 nm	0.7278	0.06767	0.5951 to 0.8604	0.0043	< 12.51
	50-100 nm	0.8451	0.05280	0.7416 to 0.9486	<0.0001	< 11.25
	100-150 nm	0.9424	0.02018	0.9028 to 0.9819	<0.0001	< 16.12
	150-200 nm	0.8509	0.06022	0.7329 to 0.9690	<0.0001	< 17.76
MCF-10A vs. MDA-MB-468	0- 50 nm	0.8333	0.05312	0.7292 to 0.9374	<0.0001	< 8.003
	50-100 nm	0.9196	0.03523	0.8505 to 0.9886	<0.0001	< 10.17
	100-150 nm	0.9615	0.01512	0.9319 to 0.9912	<0.0001	< 16.07
	150-200 nm	0.8939	0.05399	0.7881 to 0.9997	<0.0001	< 14.09
MCF-10A vs. MDA-MB-231	0- 50 nm	0.9652	0.02067	0.9247 to 1.000	<0.0001	< 5.943
	50-100 nm	0.9512	0.02577	0.9007 to 1.000	<0.0001	< 9.747
	100-150 nm	0.9988	0.001347	0.9961 to 1.000	<0.0001	< 13.13
	150-200 nm	0.9348	0.03888	0.8586 to 1.000	<0.0001	< 13.59
MCF-7 vs. SK-BR-3	0- 50 nm	0.5721	0.08278	0.4099 to 0.7344	0.3822	< 12.63
	50-100 nm	0.8170	0.06173	0.6960 to 0.9380	<0.0001	< 10.66
	100-150 nm	0.8279	0.04118	0.7471 to 0.9086	<0.0001	< 14.85
	150-200 nm	0.7749	0.05945	0.6584 to 0.8914	0.0002	< 14.80
MCF-7vs. MDA-MB-468	0- 50 nm	0.6923	0.07325	0.5487 to 0.8359	0.0174	< 6.923
	50-100 nm	0.9237	0.03646	0.8522 to 0.9952	<0.0001	< 8.998
	100-150 nm	0.8821	0.03205	0.8193 to 0.9449	<0.0001	< 14.14
	150-200 nm	0.8667	0.04806	0.7725 to 0.9609	<0.0001	< 14.29
MCF-7 vs. MDA-MB-231	0- 50 nm	0.8779	0.04884	0.7822 to 0.9737	<0.0001	< 6.004
	50-100 nm	0.9677	0.02054	0.9275 to 1.000	<0.0001	< 9.073
	100-150 nm	0.9802	0.01040	0.9598 to 1.000	<0.0001	< 12.56
	150-200 nm	0.9230	0.03887	0.8468 to 0.9992	<0.0001	< 12.99
SK-BR-3 vs. MDA-MB-468	0- 50 nm	0.6667	0.07702	0.5157 to 0.8176	0.0434	< 7.073
	50-100 nm	0.7879	0.06882	0.6530 to 0.9228	0.0008	< 8.965
	100-150 nm	0.6364	0.05580	0.5270 to 0.7458	0.0182	< 10.69
	150-200 nm	0.6386	0.07718	0.4873 to 0.7898	0.0839	< 13.80
SK-BR-3 vs. MDA-MB-231	0- 50 nm	0.8678	0.05247	0.7649 to 0.9706	<0.0001	< 5.993
	50-100 nm	0.8992	0.04055	0.8197 to 0.9787	<0.0001	< 8.427
	100-150 nm	0.8235	0.03990	0.7453 to 0.9017	<0.0001	< 10.88
	150-200 nm	0.8095	0.5989	0.6921 to 0.9269	0.0001	< 11.35
MDA-MB-468 vs. MDA-MB-231	0- 50 nm	0.7258	0.07389	0.5809 to 0.8706	0.0068	< 5.647
	50-100 nm	0.6334	0.07853	0.4795 to 0.7873	0.1005	< 6.011
	100-150 nm	0.6812	0.05110	0.5810 to 0.7813	0.0011	< 12.87
	150-200 nm	0.7317	0.07329	0.5880 to 0.8753	0.0054	< 9.190

Table S4. Detailed values of osmotic pressure obtained from the ROC analysis in paired sEV subgroups with different size range in distinguishing sEVs derived from different cell lines.

sEV source	Size range of the sEVs	Area	St.d error	95% confidence interval	P value	Cut off value
MCF-10A vs. MCF-7	0- 50 nm	0.5731	0.07724	0.4217 to 0.7245	0.3490	> 0.2170
	50-100 nm	0.7253	0.06586	0.5962 to 0.8543	0.0018	> 0.07188
	100-150 nm	0.6180	0.05513	0.5100 to 0.7261	0.0401	> 0.03215
	150-200 nm	0.6765	0.07522	0.5291 to 0.8240	0.0256	> 0.01835
MCF-10A vs. SK-BR-3	0- 50 nm	0.6139	0.07784	0.4613 to 0.7665	0.1535	> 0.05126
	50-100 nm	0.9583	0.02648	0.9064 to 1.000	<0.0001	> 0.1079
	100-150 nm	0.9710	0.01466	0.9423 to 0.9997	<0.0001	> 0.06854
	150-200 nm	0.9286	0.04509	0.8402 to 1.000	<0.0001	> 0.03038
MCF-10A vs. MDA-MB-468	0- 50 nm	0.8192	0.05813	0.7053 to 0.9332	<0.0001	> 0.2773
	50-100 nm	0.9779	0.01427	0.9499 to 1.000	<0.0001	> 0.1131
	100-150 nm	0.9899	0.006028	0.9781 to 1.000	<0.0001	> 0.06838
	150-200 nm	0.8835	0.05672	0.7723 to 0.9947	<0.0001	> 0.03137
MCF-10A vs. MDA-MB-231	0- 50 nm	0.9623	0.02306	0.9171 to 1.000/	<0.0001	> 0.3402
	50-100 nm	0.9975	0.003108	0.9914 to 1.000	<0.0001	> 0.2071
	100-150 nm	0.9969	0.002527	0.9920 to 1.000	<0.0001	> 0.1061
	150-200 nm	0.9438	0.03494	0.8754 to 1.000	<0.0001	> 0.03065
MCF-7 vs. SK-BR-3	0- 50 nm	0.5545	0.08423	0.3894 to 0.7196	0.5091	> 0.3000
	50-100 nm	0.8661	0.05110	0.7659 to 0.9662	<0.0001	> 0.1540
	100-150 nm	0.9743	0.01475	0.9454 to 1.000	<0.0001	> 0.07828
	150-200 nm	0.8680	0.04922	0.7715 to 0.9644	<0.0001	> 0.03032
MCF-7vs. MDA-MB-468	0- 50 nm	0.8007	0.06341	0.6834 to 0.9320	0.0001	> 0.2867
	50-100 nm	0.9318	0.03602	0.8612 to 1.000	<0.0001	> 0.2500
	100-150 nm	0.9866	0.008317	0.9703 to 1.000	<0.0001	> 0.08577
	150-200 nm	0.8291	0.06165	0.7083 to 0.9499	<0.0001	> 0.03183
MCF-7 vs. MDA-MB-231	0- 50 nm	0.9816	0.01757	0.9472 to 1.000	<0.0001	> 0.3708
	50-100 nm	1.000	0	1.000 to 1.000	<0.0001	> 0.2383
	100-150 nm	0.9985	0.001666	0.9952 to 1.000	<0.0001	> 0.08602
	150-200 nm	0.9040	0.04670	0.8125 to 0.9956	<0.0001	> 0.03110
SK-BR-3 vs. MDA-MB-468	0- 50 nm	0.7388	0.07275	0.5962 to 0.8814	0.0038	> 0.3557/
	50-100 nm	0.8030	0.07491	0.6562 to 0.9499	0.0004	> 0.2807
	100-150 nm	0.8312	0.04223	0.7485 to 0.9140	<0.0001	> 0.1453
	150-200 nm	0.5600	0.08263	0.3980 to 0.7220	0.4543	> 0.05711
SK-BR-3 vs. MDA-MB-231	0- 50 nm	0.9185	0.04513	0.8300 to 1.000	<0.0001	> 0.3653/
	50-100 nm	0.9785	0.01501	0.9491 to 1.000	<0.0001	> 0.2728
	100-150 nm	0.9395	0.02385	0.8927 to 0.9862	<0.0001	> 0.1373
	150-200 nm	0.7188	0.07710	0.5676 to 0.8699	0.0070	> 0.06221
MDA-MB-468 vs. MDA-MB-231	0- 50 nm	0.7441	0.06970	0.6075 to 0.8808	0.0034	> 0.3664
	50-100 nm	0.7595	0.06726	0.6277 to 0.8914	0.0014	> 0.5684
	100-150 nm	0.7385	0.04760	0.6452 to 0.8318	<0.0001	> 0.2249
	150-200 nm	0.6917	0.07920	0.5364 to 0.8469	0.0214	> 0.08583

Table S5. Detailed values of stiffness obtained from the ROC analysis in paired sEV subgroups with different size range in distinguishing sEVs derived from different cell lines.

sEV source	Size range of the sEVs	Area	St.d error	95% confidence interval	P value	Cut off value
MCF-10A vs. MCF-7	0- 50 nm	0.7833	0.06354	0.6588 to 0.9079	0.0003	> 0.01399
	50-100 nm	0.7404	0.06247	0.6179 to 0.8628	0.0008	> 0.01350
	100-150 nm	0.6030	0.05659	0.4921 to 0.7139	0.0733	> 0.01020
	150-200 nm	0.6706	0.07320	0.5271 to 0.8141	0.0310	> 0.008050
MCF-10A vs. SK-BR-3	0- 50 nm	0.9090	0.03899	0.8326 to 0.9855	<0.0001	> 0.01808
	50-100 nm	0.8360	0.05785	0.7226 to 0.9494	<0.0001	> 0.009950
	100-150 nm	0.7444	0.04789	0.6505 to 0.8382	<0.0001	> 0.01240
	150-200 nm	0.6366	0.07937	0.4811 to 0.7922	0.0957	> 0.008100
MCF-10A vs. MDA-MB-468	0- 50 nm	0.9974	0.003513	0.9906 to 1.000	<0.0001	> 0.02235
	50-100 nm	0.8974	0.04098	0.8171 to 0.9778	<0.0001	> 0.01450
	100-150 nm	0.8289	0.03858	0.7533 to 0.9046	<0.0001	> 0.01250
	150-200 nm	0.6965	0.07559	0.5484 to 0.8447	0.0197	> 0.006606
MCF-10A vs. MDA-MB-231	0- 50 nm	0.9703	0.01909	0.9329 to 1.000	<0.0001	> 0.02215
	50-100 nm	0.9214	0.03356	0.8557 to 0.9872	<0.0001	> 0.01510
	100-150 nm	0.8572	0.03365	0.7912 to 0.9231	<0.0001	> 0.01010
	150-200 nm	0.9701	0.02497	0.9212 to 1.000	<0.0001	> 0.008022
MCF-7 vs. SK-BR-3	0- 50 nm	0.7885	0.06492	0.6612 to 0.9157	0.0005	> 0.01808/
	50-100 nm	0.6027	0.07979	0.4463 to 0.7591	0.2053	> 0.01450
	100-150 nm	0.6442	0.05622	0.5340 to 0.7544	0.0155	> 0.01240
	150-200 nm	0.5395	0.07676	0.3890 to 6900	0.5973	> 0.008450
MCF-7vs. MDA-MB-468	0- 50 nm	0.9822	0.01385	0.9551 to 1.000	<0.0001	> 0.02445
	50-100 nm	0.7045	0.07333	0.5608 to 0.8483	0.0138	> 0.01450
	100-150 nm	0.7421	0.04905	0.6459 to 0.8382	<0.0001	> 0.01150
	150-200 nm	0.5752	0.07918	0.4200 to 0.7303	0.3303	> 0.009750
MCF-7 vs. MDA-MB-231	0- 50 nm	0.9239	0.03599	0.8534 to 0.9944	<0.0001	> 0.02365
	50-100 nm	0.8041	0.05638	0.6936 to 0.9147	<0.0001	> 0.02035
	100-150 nm	0.7674	0.04625	0.6767 to 0.8580	<0.0001	> 0.01010
	150-200 nm	0.9154	0.03522	0.8464 to 0.9844/	<0.0001	> 0.009050
SK-BR-3 vs. MDA-MB-468	0- 50 nm	0.9183	0.03711	0.8455 to 0.9910	<0.0001	> 0.02604
	50-100 nm	0.6184	0.08408	0.4536 to 0.7832	0.1693	> 0.01700
	100-150 nm	0.6075	0.05611	0.4976 to 0.7175	0.0626	> 0.01550
	150-200 nm	0.5950	0.08013	0.4379 to 0.7521	0.2361	> 0.005937
SK-BR-3 vs. MDA-MB-231	0- 50 nm	0.8071	0.06828	0.6732 to 0.9409	0.0003	> 0.02925
	50-100 nm	0.7272	0.06941	0.5911 to 0.8632	0.0041	> 0.02035
	100-150 nm	0.6316	0.05439	0.5250 to 0.7382	0.0199	> 0.01010
	150-200 nm	0.8579	0.05006	0.7598 to 0. 9560	<0.0001	> 0.008893
MDA-MB-468 vs. MDA-MB-231	0- 50 nm	0.5970	0.09425	0.4123 to 0.7817	0.2453	> 0.03441
	50-100 nm	0.6202	0.07902	0.4654 to 0.7751	0.1388	> 0.02035
	100-150 nm	0.5236	0.05633	0.4132 to 0.6340	0.6711	> 0.01010
	150-200 nm	0.7892	0.06607	0.6597 to 0.9187	0.0005	> 0.008022

Reference

[S1] W. Rawicz, K. C. Olbrich, T. McIntosh, D. Needham, E. Evans, *Biophys. J* **2000**, *79*, 328.

[S2] Y. C. B. Fung, P. Tong, *Biophysical Journal* **1968**, *8*, 175.

[S3] D. Vorselen, F. C. MacKintosh, W. H. Roos, G. J. L. Wuite, *ACS Nano* **2017**, *11*, 2628.

[S4] P. B. Canham, *Journal of theoretical biology* **1970**, *26*, 61.

[S5] W. Helfrich, *Zeitschrift für Naturforschung C* **1973**, *28*, 693.

[S6] A. Calo, D. Reguera, G. Oncins, Persuy, M-A., Sanz, G., Lobasso, S., Corcelli, A., Pajot-Augy, E., Gomila, G., *Nanoscale* **2014**, *6*, 2275.
